# Mitochondrial perturbation in immune cells enhances cell-mediated innate immunity in *Drosophila*

**DOI:** 10.1186/s12915-024-01858-5

**Published:** 2024-03-13

**Authors:** Laura Vesala, Yuliya Basikhina, Tea Tuomela, Anssi Nurminen, Emilia Siukola, Pedro F. Vale, Tiina S. Salminen

**Affiliations:** 1 Faculty of Medicine and Health Technology, Tampere University, Tampere, Finland; 2 Department of Molecular Biology, Umeå University, Umeå, Sweden; 3 Institute of Ecology and Evolution, School of Biological Sciences, University of Edinburgh, Edinburgh, UK

**Keywords:** Aerobic glycolysis, Hemocyte, Infection, Lamellocyte, *Leptopilina boulardi*, Mitochondrial membrane potential, Mitochondrial unfolded protein response, Oxidative phosphorylation, Reactive oxygen species, RNA sequencing, *UQCR-C1*

## Abstract

**Background:**

Mitochondria participate in various cellular processes including energy metabolism, apoptosis, autophagy, production of reactive oxygen species, stress responses, inflammation and immunity. However, the role of mitochondrial metabolism in immune cells and tissues shaping the innate immune responses are not yet fully understood. We investigated the effects of tissue-specific mitochondrial perturbation on the immune responses at the organismal level. Genes for oxidative phosphorylation (OXPHOS) complexes cI-cV were knocked down in the fruit fly *Drosophila melanogaster*, targeting the two main immune tissues, the fat body and the immune cells (hemocytes).

**Results:**

While OXPHOS perturbation in the fat body was detrimental, hemocyte-specific perturbation led to an enhanced immunocompetence. This was accompanied by the formation of melanized hemocyte aggregates (melanotic nodules), a sign of activation of cell-mediated innate immunity. Furthermore, the hemocyte-specific OXPHOS perturbation induced immune activation of hemocytes, resulting in an infection-like hemocyte profile and an enhanced immune response against parasitoid wasp infection. In addition, OXPHOS perturbation in hemocytes resulted in mitochondrial membrane depolarization and upregulation of genes associated with the mitochondrial unfolded protein response.

**Conclusions:**

Overall, we show that while the effects of mitochondrial perturbation on immune responses are highly tissue-specific, mild mitochondrial dysfunction can be beneficial in immune-challenged individuals and contributes to variation in infection outcomes among individuals.

**Supplementary Information:**

The online version contains supplementary material available at 10.1186/s12915-024-01858-5.

## Background

Eliciting an effective immune response requires coordinated metabolic changes, including the mobilization and allocation of energy reserves. Mitochondria have a central role in the metabolic control of immune responses as they produce ATP via oxidative phosphorylation (OXPHOS), a primary function that is conserved in eukaryotes. The OXPHOS system is composed of five protein complexes (cI-cV), located at the inner membrane of the double-membraned mitochondria. Each of these complexes is comprised of subunits encoded both by the nuclear and mitochondrial genome (mtDNA), except cII which only contains nuclear encoded proteins. Disruption of any of these complexes can cause mitochondrial dysfunction, which is involved in numerous pathologies including neurodegenerative diseases, metabolic disorders such as diabetes, myopathies [1, 2], chronic fatigue syndrome [3], multiple sclerosis [4], mood disorders [5] and intestinal diseases such as irritable bowel syndrome [6]. During the past decade, the role of mitochondria in innate and adaptive immune responses has been increasingly studied and mitochondria have been shown to participate in immune responses in several ways, for example by controlling apoptosis, producing various signaling molecules, metabolites and reactive oxygen species (ROS) [7–12]. Furthermore, once it enters the cytoplasm or extracellular space, mtDNA acts as a damage-associated molecular pattern (DAMP), initiating the innate immune response [13].

Mitochondrial and nuclear mutations leading to a disruption of mitochondrial function are expected to be harmful in the context of immunity, and indeed, the evidence from systemic mitochondrial disorders suggests that such disruptions lead to immunodeficiency (reviewed in [14]). For example, in the fruit fly *Drosophila melanogaster* model, an incompatibility in the interaction between the mt-tRNA_Tyr_ and mt-tyrosyl-tRNA synthetase variants encoded by the mitochondrial and the nuclear genomes, respectively, resulted in compromised OXPHOS, and weakened the innate immune response against a bacterial pathogen [15]. However, as tissues vary in their energetic demands, disruption of mitochondrial function can cause distinct effects on immune responses depending on the affected tissue. Importantly, immune responses have not been studied extensively in connection with mitochondrial dysfunction, and the impact of tissue-specific mitochondrial metabolism on the immune response is currently not well understood. *D. melanogaster* has long been used as a model for studying innate immunity, due to the conservation of signaling cascades and effector molecules between the fly and humans (reviewed in [16]). Two tissue types crucial for a fly immune response both at larval and adult stages are the fat body, functionally akin to mammalian liver and adipose tissue [17], and the hemocytes, the fly immune cells [18]. The fat body, as the main glycogen storage organ, is integral in providing energy for the immune response. The fat body also produces antimicrobial peptides (AMPs), mainly regulated by two NF-κB pathways, Toll and Imd, during the humoral immune response against microbes [19]. Cell-mediated innate immune responses rely on the hemocytes, which are produced in two waves during development: embryonic and larval hematopoiesis [20]. At the larval stages, the hemocyte system consists of phagocytic cells called plasmatocytes, which are functionally similar to mammalian macrophages [21], and an immune-inducible hemocyte type called the lamellocyte [22, 23]. Lamellocytes are large, discoidal cells able to encapsulate pathogens that are too large for phagocytosis, such as parasitoids. A third hemocyte type, the crystal cell, functions mainly in wound healing and is present in relatively low numbers in larvae and adults [24, 25]. All larval hemocyte types can be produced from progenitor cells in the lymph gland [26], but embryonic plasmatocytes also persist in the larval stage and contribute to the larval hemocyte pool [20, 27]. In addition, it has been shown that lamellocytes can transdifferentiate from plasmatocytes/progenitor cells outside the lymph gland [28–30].

To shed light on how immune-tissue specific mitochondrial dysfunction affects innate immune responses, we utilized the *Drosophila* model, silencing selected genes from each of the five OXPHOS complexes in the fat body and hemocytes. While knocking down OXPHOS genes in the fat body was harmful, OXPHOS perturbation in hemocytes induced immune activation of hemocytes and provided protection against parasitoid wasp infection. We present a potential mechanism for these effects in hemocytes and discuss the impact of OXPHOS disruption on the variation in infection outcomes.

## Results

### OXPHOS perturbation ubiquitously and in the fat body, but not in hemocytes, reduces the viability of the flies

**Fig. 1:**
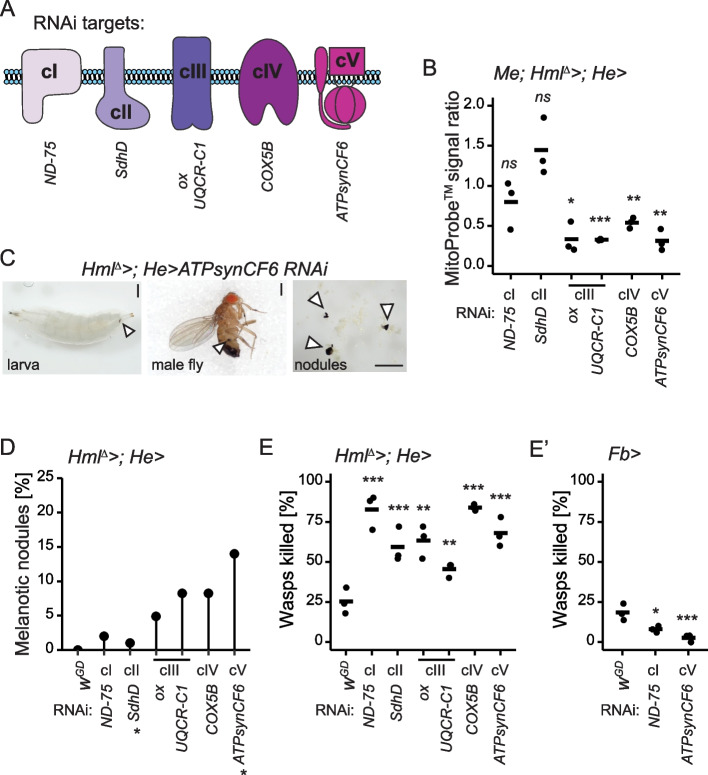
Knockdown of the OXPHOS complex genes in hemocytes affects the mitochondrial membrane potential, causes melanotic nodules and enhances the immune competence.** A** A schematic representation of oxidative phosphorylation (OXPHOS) complexes and the complex-specific knockdown target genes: *ND-75* (*NADH dehydrogenase (ubiquinone*) *75 kDa subunit*); *SdhD* (*Succinate dehydrogenase, subunit D*); *ox* (*oxen*); *UQCR-C1* (*Ubiquinol-cytochrome c reductase core protein 1*); *COX5B* (*Cytochrome c oxidase subunit 5B*); *ATPsynCF6* (*ATP synthase, coupling factor 6*). **B** Mitochondrial membrane potential was measured as a ratio of the MitoProbe™ TMRM signal intensity detected in the OXPHOS knockdown plasmatocytes to the signal detected in control plasmatocytes (*n* = 3, 2000–3500 *eater-GFP*-positive plasmatocytes per replicate). The data were analyzed using one sample t-test. **C** Examples of melanotic nodules found from hemocyte-targeted cV knockdown animals. Nodules are marked with arrowheads. Scale bars 500 µm. **D** Quantification of melanotic nodules detected in the hemocyte-targeted OXPHOS gene knockdown larvae (*n* = 100). * = 1% of the driverless background control larvae had melanotic nodules. **E** Mean percentage of melanized wasp eggs/larvae found in the controls and in larvae with OXPHOS knockdown in hemocytes (*n* = 150). **E’** Melanization response to wasp infection in the animals with *ND-75* and *ATPsynCF6* knockdown in the fat body (*n* = 150). The data were analyzed using logistic regression with a binomial distribution with replication as a random factor. ns = not significant, * *p* < 0.05, ** *p* < 0.01, *** *p* < 0.001. (Schematic Fig. 1A modified from [10])

To test how severely mitochondrial perturbation affects the host, we individually silenced genes from OXPHOS complexes I-V (cI-cV) in the immune tissues (fat body and hemocytes) as well as ubiquitously (whole animal). From each of the five OXPHOS complexes, one to two genes were targeted in the knockdown experiments (Fig. 1A, Additional file 1: Table S1 & S2). Ubiquitous knockdown (*da-GAL4*) of individual OXPHOS genes resulted in a severe larval developmental delay and was eventually lethal (Additional file 1: Table S3). In the case of the fat body-specific gene silencing (*Fb-GAL4*) the effects varied from developmental delay to lethality, depending on the OXPHOS complex. Fat body-specific knockdown of *ND-75* (cI) and *SdhD* (cII) resulted in a development delay but viable adults, whereas knockdown of *ox* (cIII), *UQCR-C1* (cIII), *COX5B* (cIV) and *ATPsynCF6* (cV) were partially or fully lethal at the pupal stage (Additional file 1: Table S3). In contrast, knocking down the OXPHOS genes in hemocytes (*Hml*^*Δ*^*-GAL4; He-GAL4*) did not affect the development time or eclosion of flies.

Taken together, the effect of OXPHOS perturbation varied according to the affected OXPHOS complex and the target tissue. Fat body-specific knockdown of cI-cII genes resulted in a milder effect on the viability than cIII-cV gene knockdown, while hemocyte-specific knockdown did not affect the development time or eclosion of the flies. Given the central role of hemocytes in cell-mediated innate immunity, we focused on the effects of OXPHOS perturbation in larval hemocytes on cellular innate immune response.

### Knockdown of OXPHOS genes in hemocytes decreases the mitochondrial membrane potential and induces the formation of melanotic nodules

First, we verified that the RNAi constructs efficiently silenced the expression of their target genes in hemocytes. *ND-75*, *SdhD*, *UQCR-C1* and *ATPsynCF6* expression was significantly reduced, ranging from 57 to 91% of the control levels in females (Additional File 2: Fig. S1A) and from 69 to 94% in males (Additional File 2: Fig. S1A’). We were not able to measure the RNAi efficiencies of *ox* and *COX5B*, as the RNAi constructs’ hairpin sequence covers most of the gene length, leaving no space to design appropriate reverse transcription quantitative real-time PCR (RT-qPCR) primers for the remaining gene sequence.

To verify that the silencing of the OXPHOS genes resulted in mitochondrial perturbation, we measured the mitochondrial membrane potential (ΔΨm) as a readout of mitochondrial activity. ΔΨm was measured from plasmatocytes using a membrane potential sensitive MitoProbe dye. As a control, hemocytes were treated with carbonyl cyanide 3-chlorophenylhydrazone (CCCP), an uncoupler of oxidative phosphorylation in mitochondria causing a drastic drop in the ΔΨm (Additional File 2: Fig. S1B). We found that cIII-cV knockdown hemocytes had significantly lower MitoProbe signal intensity than the untreated control hemocytes, corresponding to a 40–80% decrease in ΔΨm (Fig. 1B, Additional File 2: Fig. S1B). In contrast, knockdown of cI-cII did not have a significant effect on the MitoProbe signal (Fig. 1B).

Next, we investigated if silencing of the OXPHOS genes alters the cell-mediated innate immune response of the host. Salminen et al. [31] showed that a mtDNA-encoded OXPHOS cIII gene *cytochrome b* (*mt:cyt-b*) variant was associated with the formation of melanotic nodules in *Drosophila*. Melanotic nodules are melanized hemocyte aggregates and are indicative of a pre-activated cell-mediated immune system [32]. We found that silencing any of the OXPHOS genes in hemocytes led to the formation of melanotic nodules to varying degrees, examples of which are shown in Fig. 1C. The prevalence of the nodules ranged from 2 to 14% in the GD library RNAi lines (Fig. 1D), and the finding was further confirmed, although with a milder phenotype, with an additional set of RNAi lines from the KK library with a different genetic background (Additional File 3: Fig. S2A). Again, the strongest phenotypes were observed with cIII-cV gene knockdowns (Fig. 1D). In the control animals, 0–1% of the larvae exhibited melanotic nodules (Fig. 1D).

Hemocyte-targeted OXPHOS gene knockdowns did not affect the development time or eclosion. Nevertheless, perturbation of OXPHOS decreased the mitochondrial membrane potential in hemocytes and activated cell-mediated innate immunity, which could potentially affect other life-history traits of the flies. Therefore, we tested whether cIII and cV gene knockdowns in hemocytes had an effect on the lifespan of the flies. While the male longevity was not affected by the cIII or cV knockdown, females with cIII knockdown exhibited significantly decreased lifespan (Additional File 3: Fig. S2B). The reduced lifespan in females upon cIII knockdown in hemocytes might suggest that there are differences in the demands for hemocyte metabolism in adult flies between the sexes.

### OXPHOS perturbation in hemocytes enhances the cell-mediated encapsulation response against parasitoid wasps

Because melanotic nodules indicate activation of the cellular innate immune response [32], we examined if OXPHOS perturbation in hemocytes affected the cell-mediated response against parasitoid wasp infection. We infected the knockdown and control larvae with *Leptopilina boulardi* parasitoid wasps and scored the percentage of larvae with a successful immune response (fully melanized wasp larvae). Knockdown of the OXPHOS genes resulted in an enhanced encapsulation response when compared to the controls (Fig. 1E). A similar trend was observed when knocking down the OXPHOS genes using the KK RNAi lines (Additional File 3: Fig. S2C), although the effect was again less pronounced than in the GD RNAi lines.

Next, we tested if OXPHOS perturbation in the fat body also resulted in an altered response to wasp infection. Fat body specific (*Fb-GAL4*) knockdown of *ND-75* (cI) had the mildest larval development delay when compared to other OXPHOS complex knockdowns, and *ATPsynCF6* (cV) knockdown had one of the most severe effects on viability (Additional file 1: Table S3). In both knockdown lines, the encapsulation response was drastically reduced when compared to the control (Fig. 1E’). Taken together, OXPHOS perturbation in the fat body had a negative impact on viability and led to an increased susceptibility to immune challenge, indicating the importance of tightly regulated mitochondrial function in this tissue. In contrast, hemocyte-targeted OXPHOS perturbation did not affect development and was beneficial for the host upon an immune challenge. This suggests that OXPHOS acts as a moderator of hemocyte function under normal conditions.

### OXPHOS perturbation in hemocytes results in immune cell activation

**Fig. 2:**
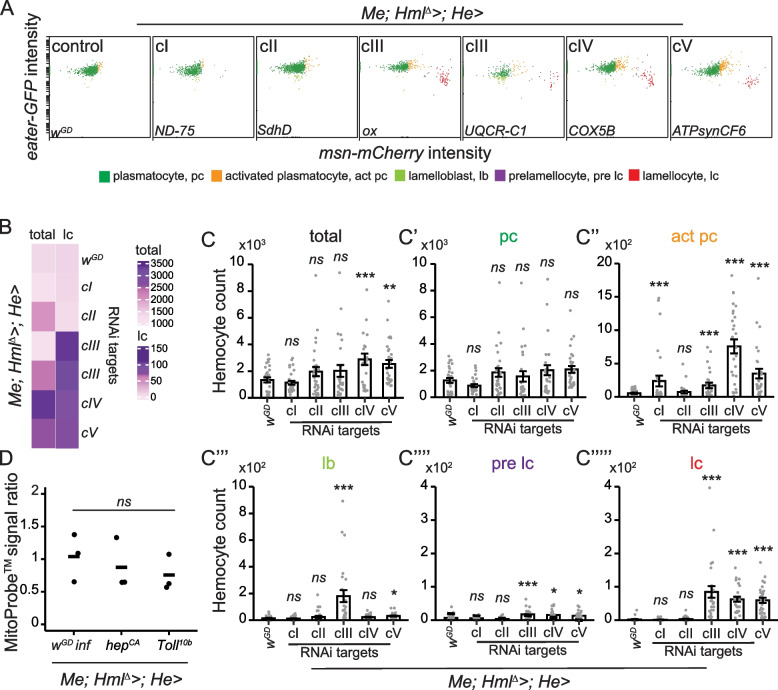
Knockdown of the OXPHOS genes in hemocytes induces immune cell activation. **A** Flow cytometry analysis of larval hemocytes using the plasmatocyte (*eater-GFP*, y-axis) and lamellocyte (*msn-mCherry,* x-axis) in vivo hemocyte reporters. While the control animals mainly had plasmatocytes (high GFP), the knockdown of various OXPHOS genes induced immune-activated hemocyte types, including activated plasmatocytes (high GFP, low mCherry) and lamellocytes (high mCherry intensity, marked in red), and occasionally also lamelloblasts (low GFP) and prelamellocytes (low GFP, low mCherry). **B** Heatmap (*n* = 30) showing the average total hemocyte and lamellocyte (lc) count in *w*^*GD*^ control and hemocyte-targeted OXPHOS knockdown samples. **C**–**C’’’’’** Quantification of total hemocytes and hemocyte types classified based on *eater-GFP* and *msn-mCherry* expression when knocking down the OXPHOS genes (*n* = 30). Total = total circulating hemocyte count, pc = plasmatocytes, act pc = activated plasmatocytes, lb = lamelloblasts, pre lc = pre-lamellocytes, lc = lamellocytes. cIII gene = *UQCR-C1*. The data on hemocyte counts were analyzed using a generalized linear model with a negative binomial distribution. Error bars indicate standard error of the mean. Asterisks indicate the statistical difference between the OXPHOS knockdowns and the control*.* **D** Mitochondrial membrane potential in plasmatocytes of wasp-infected larvae and of larvae with *hep*^*CA*^ or *Toll*^*10b*^ overexpression in hemocytes, as a ratio to the MitoProbe™ TMRM signal intensity in the control. ns = not significant, * *p* < 0.05, ** *p* < 0.01, *** *p* < 0.001

Having established a link between mitochondrial perturbation in hemocytes, formation of melanotic nodules and an enhanced immune response against parasitoid infection, we tested if silencing the OXPHOS genes in hemocytes affects hemocyte numbers or differentiation. We utilized an in vivo hemocyte reporter system (*msn-mCherry* and *eater-GFP; “Me”* for short) to distinguish hemocyte subpopulations based on the levels of the mCherry and GFP fluorescence as described in Anderl et al. [28]. As expected, in the control larvae mainly plasmatocytes were present, as shown by high levels of *eater-GFP* expression (Fig. 2A). In contrast, knocking down OXPHOS cIII, cIV and cV-related genes resulted in an immune activation of the hemocytes, with uninfected larvae producing infection-specific hemocytes, including lamellocytes, identified by high *msn-mCherry* expression and a lack of the *eater-GFP* expression (Fig. 2A-B). In addition to hemocyte activation, total hemocyte numbers were significantly increased upon cIV and cV knockdowns, without a change in basal state plasmatocyte numbers (Fig. 2C-C’), but with an increase in activated plasmatocytes (Fig. 2C’’), which are characterized by high GFP expression together with mCherry fluorescence, which is often localized in cytoplasmic foci [28]. These hemocytes are not present in high numbers in healthy larvae, but appear after wasp infestation or genetic activation of the hemocytes [28]. Numbers of lamelloblasts, the putative lamellocyte precursor cell type [28], were increased by cIII and cV knockdowns (Fig. 2C’’’), whereas prelamellocytes (Fig. 2C’’’’), and to an even greater extent lamellocytes (Fig. 2C’’’’’), were increased upon cIII, cIV and cV gene knockdowns (Fig. 2C’’’’). cI and cII knockdown had mild effects on hemocyte populations (Fig. 2A, C–C’’’’’). Knocking down the OXPHOS genes using the KK library RNAi constructs showed similar results concerning hemocyte activation (Additional File 4: Fig. S3A-A’’’’’), except for knockdown of *ND-75,* which resulted in the formation of prelamellocytes and lamellocytes (Additional File 4: Fig. S3A’’’’-A’’’’’), indicating that cI silencing may also induce lamellocyte formation in specific genetic backgrounds.

Wasp infection is known to cause both elevation in hemocyte numbers and hemocyte activation. We tested if hemocyte-specific cI, cIII or cV knockdown has additional effects on the hemocyte populations 48 h after wasp infection compared to wasp-infected controls. In the wasp-infected larvae, OXPHOS knockdown did not alter hemocyte profiles and produced similar numbers of lamellocytes as the wasp-infected control larvae (Additional File 4: Fig. S3B-B’’’’’). The exceptions were a small decrease in the numbers of activated plasmatocytes in the cIII knockdown larvae, and an increase in lamelloblast count in the cV knockdown larvae (Additional File 4: Fig. S3B’’).

Overall, hemocyte-specific OXPHOS perturbation leads to increased hemocyte counts and immune activation prior to infection. The hemocyte profile induced by wasp infection was mostly unaltered by the OXPHOS knockdown.

### Decrease in the mitochondrial membrane potential is not a universal indicator of immune cell activation

cIII-cV gene knockdowns caused a decrease in ΔΨm in plasmatocytes, while cI and cII gene knockdowns did not. In parallel, cIII-cV knockdowns led to the formation of melanotic nodules, elevated immune cell count and immune cell activation, which was not the case with the cI and cII gene knockdowns. This prompted us to test whether a decrease in the circulating plasmatocyte ΔΨm occurs when they begin to transdifferentiate into lamellocytes. We selected three model systems that are known to induce the formation of lamellocytes: parasitoid wasp infection, and the expression of constitutively active forms of *hemipterous* (*hep*^*CA*^) and *Toll* (*Toll*^*10b*^, *Tl*^*10b*^) [33]. There were no significant differences in ΔΨm compared to the control, although there was a trend for lower ΔΨm in *hep*^*CA*^ and *Tl*^*10b*^ plasmatocytes (Fig. 2D). Based on this result, we cannot conclude with certainty that a drop in ΔΨm is a common feature of hemocyte activation. However, we do not exclude the possibility that a drop in ΔΨm might be required in hemocytes attached to tissues or in those forming the melanized capsules.

### *UQCR-C1* (cIII) knockdown hemocyte transcriptomes cluster according to time point and infection status

**Fig. 3:**
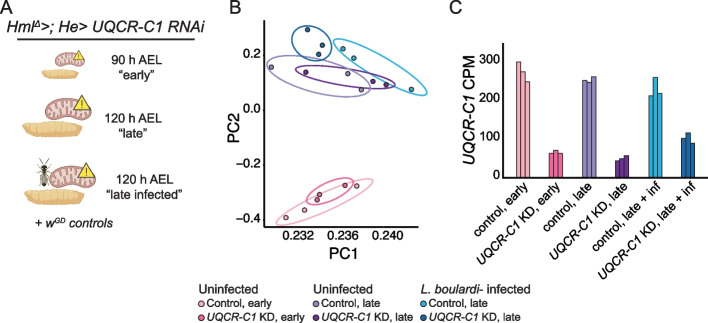
Transcriptomic analysis of *UQCR-C1* knockdown hemocytes. **A** Samples used for RNA sequencing. Hemocytes were collected from male larvae with a hemocyte-specific OXPHOS complex III knockdown (*Hml*^*Δ*^*-GAL4; He-GAL4/UAS- UQCR-C1 RNAi)* and the controls (*Hml*^*Δ*^*-GAL4; He-GAL4/w*^*GD*^*)* at 90 h after egg lay (“early” timepoint), 120 h after egg lay (“late” timepoint) as well as from wasp-infected larvae at 120 h after egg lay (“late infected” timepoint). **B** PCA plot of the RNA sequencing data (*n* = 3). **C** *UQCR-C1* expression levels in the RNA sequencing samples expressed as counts per million (CPM) showing the efficiency of the knockdown. Figure 3A created with BioRender.com

We harnessed RNA sequencing to study the mechanism behind OXPHOS perturbation that leads to immune cell activation. For this, we chose cIII gene *UQCR-C1* due to its strong effects on the immune phenotypes studied here. We sampled *UQCR-C1* knockdown hemocytes from uninfected and wasp-infected male larvae and their controls. Because *UQCR-C1* knockdown in hemocytes induces lamellocyte differentiation (Fig. 2), we first identified a timepoint at which lamellocytes are not yet present in the hemolymph, in order to capture transcriptional changes prior to and after the appearance of mature lamellocytes. Based on previous information on the lamellocyte differentiation after wasp infection [28], we profiled the hemocytes of *UQCR-C1* knockdown larvae at 90, 92 and 96 h after egg lay (AEL). While lamellocytes were detected in the circulation both 92 h and 96 h AEL, at 90 h AEL lamellocyte numbers did not differ from the age-matched control larvae (Additional File 5: Fig. S4A’’’’’ for lamellocyte counts, Additional File 5: Fig. S4A-A’’’’ for other hemocyte types). In addition, *UQCR-C1* knockdown plasmatocytes exhibited reduced ΔΨm at 90 h AEL (Additional File 5: Fig. S4B-B’), showing that the mitochondrial perturbation is present already at that timepoint. The 90 h AEL timepoint was therefore selected as the sample collection timepoint, and is hereafter referred to as the “*early*” timepoint*.* In addition, we collected *UQCR-C1* knockdown and control hemocytes from late 3rd instar larvae (approximately 120 h AEL) without and with parasitoid wasp infection (48 h post infection), referred to as “*late*” and “*late infected*”, respectively (Fig. 3A). Of note, “*late infected*” refers to a comparison between *UQCR-C1* knockdown hemocytes collected from wasp-infected larvae and control hemocytes also from wasp-infected larvae.

Principal component analysis (PCA, Fig. 3B) showed a clear clustering of the transcriptome samples along the PC2 according to timepoint and (to a lesser degree) the wasp-infection status. In addition, wasp-infected samples clustered based on their genotype (*UQCR-C1* knockdown or control) along PC1. The knockdown of *UQCR-C1* was verified based on the counts per million (CPM) values of the RNA sequencing data for all three treatment groups, with a drop in *UQCR-C1* expression to 20–45% of the control value (Fig. 3C).

### *UQCR-C1* knockdown causes upregulation of lamellocyte-enriched marker genes at both timepoints

*UQCR-C1* knockdown in hemocytes led to distinct changes in the transcriptome. A subset of genes was differentially expressed in all treatments/timepoints when *UQCR-C1* was silenced, and multiple genes were differentially regulated only at specific timepoints or upon infection (all significantly differentially expressed genes are listed in Additional File 6). Because *UQCR-C1* silencing led to lamellocyte differentiation, many differentially expressed genes were likely to be linked to lamellocyte function, especially at the late timepoint. We utilized a comprehensive list of 304 lamellocyte-enriched marker genes based on the hemocyte single-cell RNA sequencing studies compiled by Hultmark and Ando [34] and cross-referenced these genes with our early and late timepoint *UQCR-C1* samples. Indeed, many lamellocyte-enriched genes were upregulated in uninfected *UQCR-C1* knockdown samples at the late, and, interestingly, also at the early timepoint (114 and 113 genes, respectively; Additional File 7). There was a large degree of overlap in lamellocyte-enriched genes upregulated at late and early timepoint (94 genes). When comparing infected *UQCR-C1* knockdown samples to infected control samples, only 14 lamellocyte genes were upregulated. For comparison, in the wasp-infected control hemocytes, 223 lamellocyte genes were upregulated compared to the uninfected controls (Additional Files 6 and 7). Even though both early and late timepoint *UQCR-C1* knockdown hemocytes showed similar upregulation of the lamellocyte marker genes, they clustered separately based on the PCA (Fig. 3B). These data indicate that even though at the early timepoint mature lamellocytes are not yet detected in the larvae, the activation program leading to them is already evident. Next, we studied further the transcriptome profiles between *UQCR-C1* knockdown and control samples.

### *UQCR-C1* knockdown leads to gene expression changes characteristic of the mitochondrial unfolded protein response

**Fig. 4:**
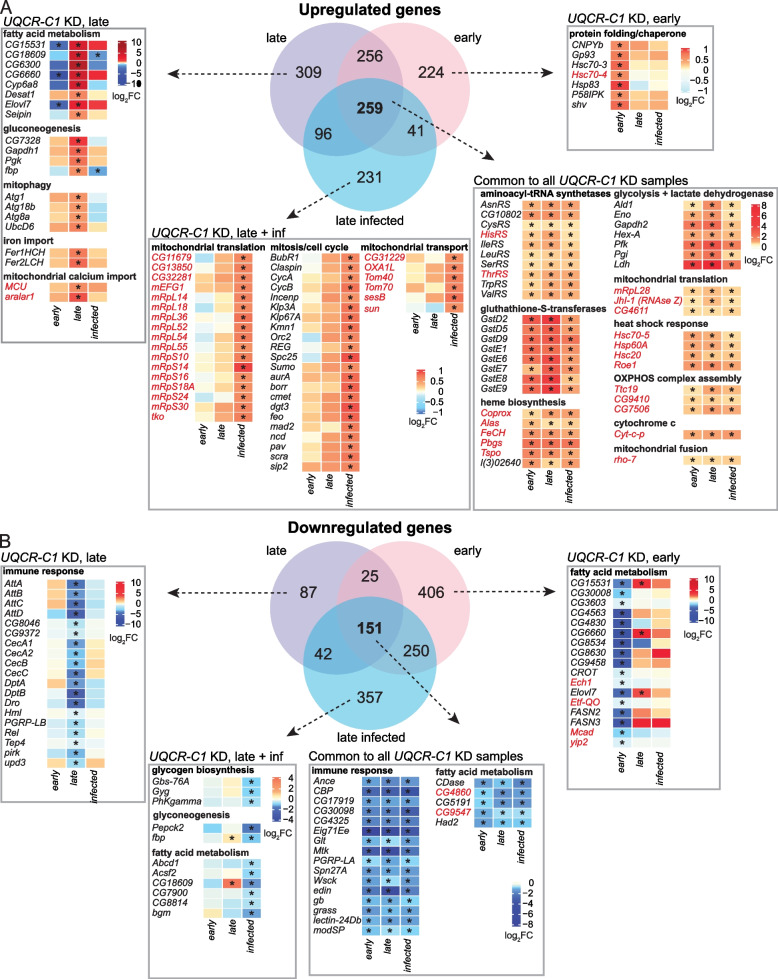
Knocking down *UQCR-C1* in hemocytes leads to changes in the hemocyte transcriptomes. Venn diagrams show genes that are upregulated (**A**) and downregulated (**B**) upon *UQCR-C1* knockdown in hemocytes, and heatmaps illustrate differential expression (as log_2_FC to *w*^*GD*^ hemocytes) of selected genes specific to all samples of *UQCR-C1* knockdown hemocytes, or unique to specific timepoint/treatment. Differentially expressed genes were grouped according to Flymine GO term pathway enrichment analysis. Asterisks indicate statistically significant difference in gene expression compared to *w*^*GD*^ control. Gene symbols written in red encode mitochondrial proteins

We utilized the Flymine [35] tool to analyze the differentially expressed genes and to construct curated lists of genes of interest based on Gene Ontology (GO) term enrichment. Significant GO terms and genes included in them are listed in Additional File 8. Genes that were consistently differentially expressed when *UQCR-C1* was knocked down were of a particular interest since those represent a core set of genes responding to *UQCR-C1* silencing, regardless of the infection status or timepoint. Across all of the timepoints and treatments, there was an upregulation of glycolytic enzymes including lactate dehydrogenase, indicating more active use of glycolysis by the cells starting already at an early timepoint prior to lamellocyte formation (Fig. 4A; Additional File 6). Many translation-related genes were also upregulated, including multiple aminoacyl-tRNA synthetases and several genes functioning in mitochondrial translation (Fig. 4A; Additional File 8). This, along with the upregulation of multiple mitochondrial genes involved in heme synthesis, OXPHOS assembly and mitochondrial fusion, might be an effort to compensate for the mitochondrial defect caused by the cIII disruption. Several heat shock response genes (Fig. 4A) and glutathione-S-transferases (Fig. 4A and Additional File 9: Fig. S5A) were upregulated, suggesting a possible stress response in the hemocytes upon OXPHOS knockdown. Of note, genes involved in the same processes (aerobic glycolysis, translation of mitochondrial proteins, heat shock response) are upregulated by the mitochondrial unfolded protein response (UPR^mt^) in the nematode *Caenorhabditis elegans* and the yeast *Saccharomyces cerevisiae* [36, 37], indicating that this phenomenon could also occur in our model. The UPR^mt^ is a cellular stress response involving a mitochondria-to-nucleus signaling pathway that induces a specific transcriptional program. The UPR^mt^ protects cells from stress that originates from mitochondrial perturbation, such as OXPHOS dysfunction, mitochondrial protein misfolding, decrease of mitochondrial membrane potential or ATP depletion [36]. Hence, it is likely that the hemocyte targeted *UQCR-C1* knockdown induces the UPR^mt^.

Among the commonly downregulated genes, the only significant GO term was the cellular compartment term “extracellular region” (Additional File 8). However, manual curation of the data revealed downregulation of multiple innate immune response-related genes in all of the *UQCR-C1* knockdown samples, including the extracellularly located *modular serine protease* (*modSP*) and *Gram-positive Specific Serine protease* (*grass*), both involved in the activation of the Toll signaling pathway (Fig. 4B; Additional File 6). As a comparison, under control conditions, wasp infection induced the upregulation of a large number of genes involved in the humoral immune response, including AMPs such as multiple *Bomanins* (also known as *Immune-induced molecules*, *IM*s), *Daisho*, *Drosomycin* (*Drs*), *Cecropin A1* (*CecA1*) and *Attacin B* (*AttB*). Furthermore, Toll pathway inducer *spatzle* (s*pz*) and its activators *Spatzle-Processing Enzyme* (*SPE*) and *easter* (*ea*) were all upregulated, as was *Toll* (*Tl*) itself (Additional Files 6 and 8). The downregulation of a set of humoral immune genes in response to *UQCR-C1* knockdown could be explained by the presence of an immuno-suppressive mechanism aimed at reducing excessive inflammation response brought on by the activation of cell-mediated innate immunity (shown as the upregulation of lamellocyte-enriched marker genes), which could be especially important in immune-challenged animals. A group of genes involved in fatty acid metabolism was also downregulated, possibly signifying that there is a switch in cell metabolism, and that the *UQCR-C1* knockdown hemocytes might rely on glycolysis for energy production.

In addition to this core set of differentially expressed genes, we looked at the changes occurring only at a certain timepoint or only when hemocytes were derived from wasp-infected larvae. At the early timepoint, knocking down *UQCR-C1* led to an upregulation of an additional set of chaperones, heat shock proteins and protein folding-related genes, both cytosolic and mitochondrial (Fig. 4A; Additional File 6). This indicates that a stress response to *UQCR-C1* knockdown is launched already at 90 h AEL. The downregulated genes included an additional group of fatty acid metabolism-related genes, again highlighting early changes in cellular metabolism.

At the late timepoint, there was an upregulation in fatty acid metabolism genes, including some that were downregulated at the early timepoint (Fig. 4A; Additional File 6). Multiple gluconeogenesis-related genes were upregulated, possibly to produce more free glucose to fuel the glycolytic pathway. Several genes connected to mitochondria and mitochondrial maintenance were upregulated, including those involved in mitophagy and mitochondrial calcium import, again suggesting a mitochondrial stress response. Among the downregulated genes was a group of innate immune response genes, mostly encoding AMPs such as several Attacins and Cecropins (Fig. 4B).

In *UQCR-C1* knockdown wasp-infected samples, compared to wasp-infected controls, a large group of genes involved in mitochondrial translation and mitochondrial transport were upregulated (Additional Files 6 and 8, Fig. 4A). As mentioned above, increased expression of genes in these groups is a feature of the UPR^mt^ [36, 37]. Another large group of genes that was upregulated was that of genes involved in cell cycle regulation. Among the downregulated genes of interest were genes related to glycogen metabolism, gluconeogenesis and fatty acid metabolism (Fig. 4B).

### OXPHOS perturbation-induced hemocyte activation is not dependent on ROS

The disruption of OXPHOS, especially complexes I and III, leads to an increased electron escape, creating increased amounts of reactive oxygen species (ROS) (reviewed in [38]). An excess of ROS can overload the antioxidant enzymes, leading to the oxidation of macromolecules [39]. We looked at the expression levels of genes involved in ROS production and detoxification in our RNA sequencing data. Superoxide, the precursor of most types of ROS, is degraded by the superoxide dismutases (Sod), which catalyze its conversion to hydrogen peroxide (H_2_O_2_). H_2_O_2_ is further broken down by Catalase (Cat) in the cytosol [40]. Expression of neither *Sod1*, *Sod2* and *Sod3,* nor *Cat,* was significantly affected by *UQCR-C1* knockdown (Additional File 9: Fig. S5A). *Immune-regulated Catalase* (*Irc*) was not affected by *UQCR-C1* knockdown in uninfected samples, but was downregulated in *UQCR-C1* knockdown hemocytes upon wasp infection when compared to wasp-infected control hemocytes (Additional File 9: Fig. S5A). In contrast, under the control conditions *Irc* was upregulated upon wasp infection (Additional File 6), suggesting a suppression of this induction in wasp-infected *UQCR-C1* knockdown hemocytes. Peroxiredoxins (Prx) are sensitive to the levels of H_2_O_2_, and function in neutralizing it and other peroxides in the cell [41]. Cytosolic *Prx2* (also known as *Jafrac1*) was consistently upregulated by *UQCR-C1* knockdown, while others were either downregulated or their expression did not change (Additional File 9: Fig. S5A). Peroxidasin (*Pxn*) was not affected, but *globin1* (*glob1*), also encoding a peroxidase, was upregulated at the early and late timepoints after *UQCR-C1* knockdown (SFig. 5A) as well as after wasp infection in control hemocytes (Additional File 6), indicating that it might be specific for activated hemocytes or lamellocytes. H_2_O_2_ can also function as a signaling molecule by oxidizing cysteine residues on proteins, and can be produced via the NOX/DUOX system on demand [42]. *NADPH oxidase* (*Nox*) and *Dual oxidase* (*Duox*) were downregulated at the early timepoint with *UQCR-C1* knockdown. Multiple *Glutathione transferase D* and *-E* (*GstD* and *GstE*) genes were upregulated at both early and late timepoints with *UQCR-C1* knockdown, and some also after wasp infection (Additional File 9: Fig. S5A). Glutathione transferases act as a general defense system against various genotoxic and cytotoxic electrophilic compounds, including ROS, by catalyzing conjugation of glutathione to these compounds [43]. As mentioned above, *Gst* expression is also a feature of the UPR^mt^. Taken together, the RNA sequencing data show some upregulation of ROS scavenging system genes, indicating that there may be a modest increase in ROS upon *UQCR-C1* knockdown in hemocytes.

To further study the connection between the immune cell activation phenotype and ROS, we used three approaches. First, ROS was measured in the OXPHOS cI, cIII and cV knockdown hemocytes using the CellROX™ green probe. While feeding antioxidant N-acetylcysteine (NAC) to the larvae reduced the levels of ROS in hemocytes (Additional File 9: Fig. S5B), knocking down the OXPHOS cI, cIII or cV did not change the average CellROX green signal intensity (Additional File 9: Fig. S5B’-C) or the proportion of CellROX-positive hemocytes (Additional File 9: Fig. S5C’). Second, we checked whether knocking down antioxidant-encoding genes in hemocytes could phenocopy the effects of *UQCR-C1* knockdown on the formation of melanotic nodules, hemocyte activation and the immune response against wasps. To this end, we knocked down genes encoding cytosolic *Sod1,* the mitochondrial *Sod2* [44, 45] or *Cat* in hemocytes. This did not induce the formation of melanotic nodules (0% prevalence in *Sod1* and *Sod2* knockdowns, 1% in *Cat* knockdown, data not plotted) or lamellocyte formation (Additional File 9: Fig. S5E’’’’’). Also, other hemocyte counts remained similar to the controls (Additional File 9: Fig. S5E-E’’’’), except for an increase in the lamelloblast count (Additional File 9: Fig. S5E’’’). Regardless of the lack of a pre-induced hemocyte activation, the melanization response against wasps was enhanced when the GD library RNAi constructs were used (Additional File 9: Fig. S5D). Third, we fed NAC to control and *UQCR-C1* knockdown larvae to see if the lamellocyte formation could be blocked by alleviating ROS pharmagologically. However, NAC feeding did not block the lamellocyte lineage hemocyte (lamelloblasts, prelamellocytes, lamellocytes) induction in *UQCR-C1* knockdown larvae (Additional File 9: Fig. S5F’’’-F’’’’’). Interestingly, NAC feeding did result in decreased total hemocyte, plasmatocyte and activated plasmatocyte counts in both control and *UQCR-C1* knockdown larvae (Additional File 9: Fig. S5F-F’’). This implies that ROS has an important function in hemocyte proliferation in general. Based on these results, the OXPHOS perturbation-mediated improvement of immune response and hemocyte activation is at least partially independent of changes in ROS production.

### Glycolysis-related genes *sima* and *Ldh* are upregulated in the OXPHOS knockdown hemocytes, but do not affect OXPHOS-induced hemocyte activation

**Fig. 5:**
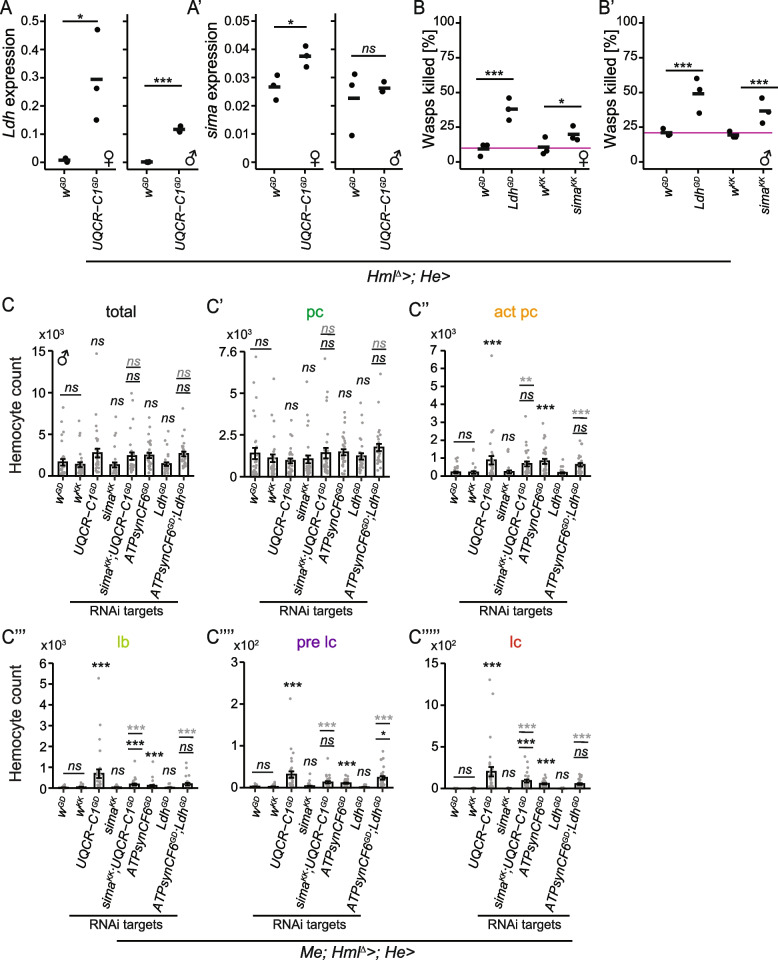
Knocking down *Ldh* or *sima* in hemocytes enhances wasp encapsulation but does not affect hemocyte activation or differentiation. **A** *Ldh* and (**A’**) *sima* expression relative to *His3.3B* in control and in *UQCR-C1* knockdown hemocytes measured by RT-qPCR (*n* = 3). **B**-**B’** The mean percentage of melanized wasp larvae found in control larvae (*w*^*GD*^ or* w*^*KK*^ as background control for *sima* knockdown) and in larvae with either *Ldh* or *sima* knockdown in hemocytes (*n* = 150). **C**–**C’’’’’** Quantification of total hemocytes and of hemocyte types classified based on *eater-GFP* and *msn-mCherry* expression in controls, in *UQCR-C1* and *sima* single knockdown, as well as in *sima; UQCR-C1* and *Ldh; ATPsynCF6* double knockdowns (*n* = 30). Data from male larvae are presented. The background strains (*w*^*GD*^ and* w*.^*KK*^) did not differ from each other in their hemocyte profiles (underlined “ns” above them). Not underlined significance symbols above the samples indicate their difference to the controls. In the case of the double knockdowns, lower underlined symbol indicates the difference to either *UQCR-C1* or *ATPsynC6* single knockdowns and upper symbol in grey the difference to the controls. Total = total circulating hemocyte count, pc = plasmatocytes, act pc = activated plasmatocytes, lb = lamelloblasts, pre lc = pre-lamellocytes, lc = lamellocytes. The data were analyzed using a generalized linear model with a negative binomial distribution. ns = not significant, * *p* < 0.05, ** *p* < 0.01, *** *p* < 0.001

Several genes related to aerobic glycolysis were upregulated when *UQCR-C1* was knocked down, in both the early and late RNA sequencing hemocyte samples (Fig. 4A). Based on this, we studied whether the metabolic switch from mitochondrial OXPHOS to cytosolic aerobic glycolysis is required and/or sufficient for the immune activation seen in *UQCR-C1* (and other OXPHOS complex) knockdown hemocytes. We first studied the expression patterns and knockdown effects of two genes which have been shown to be upregulated when hemocytes shift to utilizing aerobic glycolysis, *similar* (*sima,* the *Drosophila* homolog for *Hif1α*) and its target gene *Lactate dehydrogenase* (*Ldh*) [46]. Based on the transcriptome data, *Ldh* was upregulated at both early and late timepoints in *UQCR-C1* knockdown hemocytes (4.8 and 8.7 log_2_ FC, respectively). *sima* showed a similar trend but without statistical significance. We measured the expression of *Ldh* and *sima* in *UQCR-C1* knockdown hemocytes from both male and female larvae with RT-qPCR to see if the *UQCR-C1* knockdown effect is the same in both sexes. *Ldh* was upregulated in both sexes (Fig. 5A). However, *sima* was significantly upregulated upon *UQCR-C1* knockdown in females, while the male data resembled the RNA sequencing result; on average slight, but statistically not significant upregulation (Fig. 5A'). RT-qPCR was also harnessed to measure *Ldh* and *sima* levels in the hemocytes that had cI, cII, cIII (*ox*) or cV genes knocked down. While *sima* expression was unaffected, or even reduced in the case of *ND-75* and *ATPsynCF6* knockdown in males (Additional File 10: Fig. S6A-A'), *Ldh* was upregulated in hemocytes with *ox* and *ATPsynCF6* knockdown, and a similar trend was seen with the *ND-75* knockdown males (Additional File 10: Fig. S6B-B'). Again, cII gene *SdhD* knockdown, which has been an outlier throughout our studies, did not affect the expression of *sima* or *Ldh* (Additional File 10: Fig. S6A-B').

Hemocyte-targeted knockdown of *sima* has been shown to lead to failed increase of glycolytic gene expression (including *Ldh*) and to failed increase in Ldh enzyme activity in glycolysis-inducing conditions in *Drosophila* [46]. To further study if a metabolic switch to aerobic glycolysis in the OXPHOS knockdown hemocytes is the driving force in the lamellocyte formation and an enhanced cell-mediated immune response to wasps, we knocked down *sima* and *Ldh* in hemocytes. The expression of both genes was, in general, very low in uninduced hemocytes. However, in uninduced hemocytes both genes showed a trend towards lower expression in the knockdowns compared to the control (Additional File 10: Fig. S6C-C’). To further verify the efficacy of *sima* knockdown, we measured the expression of the Sima target *Ldh* in *sima* knockdown hemocytes. There was a trend towards reduced *Ldh* expression in *sima* knockdown hemocytes, but due to the high variability of *Ldh* expression in the control hemocytes, this difference was not statistically significant (Additional File 10: Fig. S6D).

Next, we measured the production of the reduced form of nicotinamide adenine dinucleotide (NADH) as a readout of Ldh activity in control and *Ldh* knockdown hemocytes. Again, the control samples had variable NADH values, while the *Ldh* knockdown samples showed less variation and generally lower NADH values (Additional File 10: Fig. S6E). The difference between treatments remained statistically not significant. As there was a trend towards a reduction in the expression of *sima* and *Ldh* in the knockdown lines*,* and also in the NADH levels after *Ldh* knockdown in hemocytes of the uninfected larvae, we moved on to perform further experiments with these knockdown lines.

Surprisingly*, sima* and *Ldh* knockdown in hemocytes led to an improved encapsulation response against wasps in both sexes (Fig. 5B-B'). Next, we aimed to find out if these knockdowns affect the hemocyte profiles, and whether the simultaneous knockdown of *sima* or *Ldh* combined with the OXPHOS gene knockdown (*UQCR-C1* KD + *sima* KD and *ATPsynCF6* KD + *Ldh KD*) is able to alter the hemocyte profile caused by the OXPHOS perturbation. For this, we performed an experiment with single and double knockdowns. Neither *sima* nor *Ldh* knockdowns alone resulted in increased hemocyte counts or hemocyte activation in uninfected animals (Fig. 5C-C’’’’’ for males and Additional File 10: Fig. S6F-F’’’’’ for females). Furthermore, the hemocyte population analyses showed that activated plasmatocytes, prelamellocytes and lamellocytes were still present at similar levels as in single *UQCR-C1* and *ATPsynCF6* knockdowns, both in male (Fig. 5C-C’’’’’) and female (Additional File 10: Fig. S6C-C’’’’’) larvae.

Finally, we measured the levels of the storage sugar glycogen and the main circulating sugar at larval stages, trehalose, in cI, cIII and cV knockdown larvae. In adult flies, Sima-induced aerobic glycolysis in hemocytes has been shown to be accompanied with a decrease in glycogen and an increase in circulating glucose, the main sugar in adults [46]. Glycogen levels in the OXPHOS knockdown larvae did not significantly differ from the controls (Additional File 11: Fig. S7A-A'). The level of hemolymph trehalose was generally unchanged, although increased in females when *ND-75* was silenced in hemocytes, and decreased in male *ox* knockdown larvae (Additional File 11: Fig. S7B-B’). Altogether, these results indicate that a switch to glycolytic metabolism alone is not sufficient to explain the hemocyte activation launched by the OXPHOS gene knockdown and subsequent decrease in mitochondrial membrane potential.

## Discussion

**Fig. 6:**
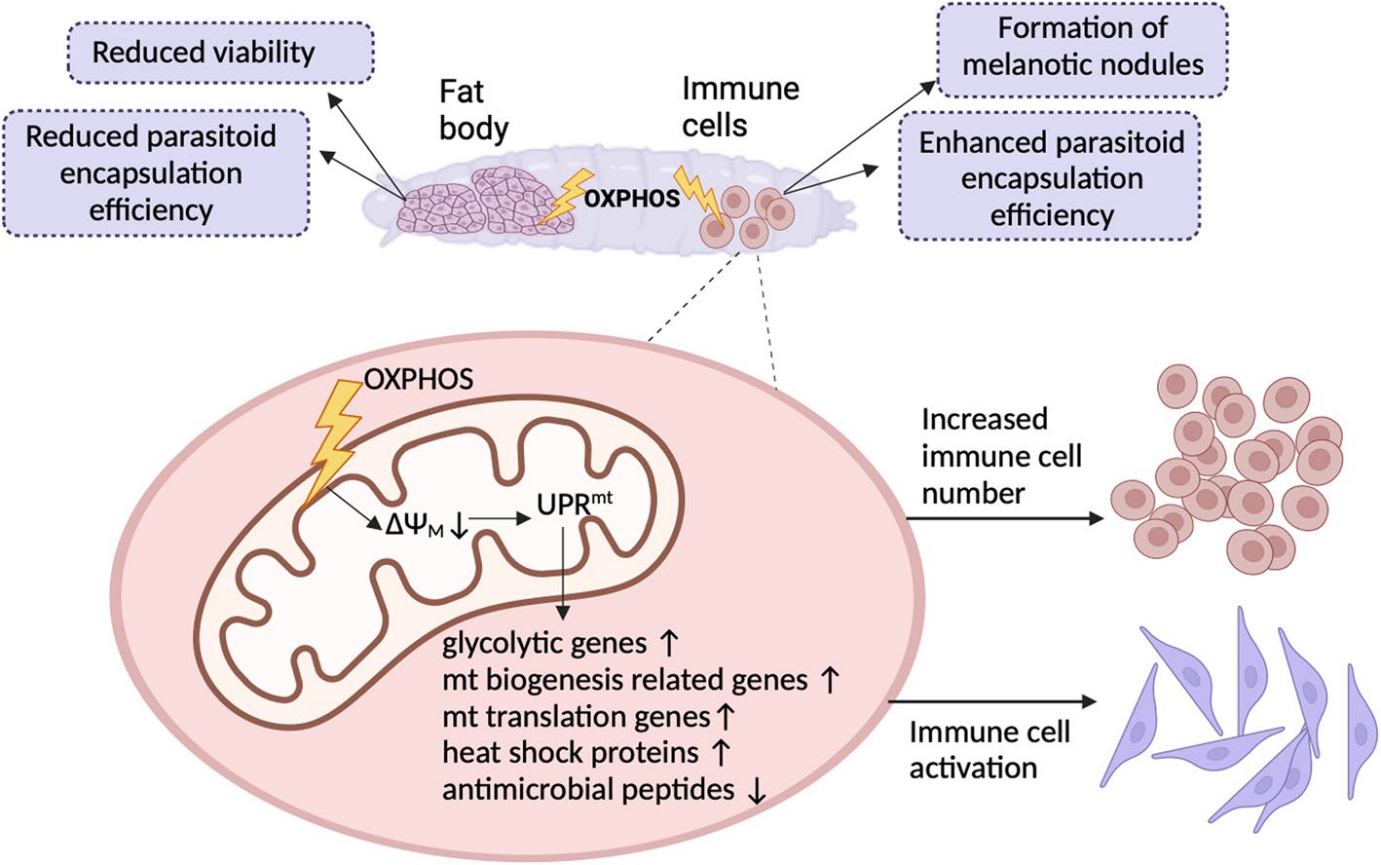
Summary of the effects of OXPHOS perturbation on cell mediated innate immunity. OXPHOS disruption in hemocytes leads to a loss of mitochondrial membrane potential (ΔΨ_m_). This likely triggers the mitochondrial unfolded protein response (UPR^mt^) in the cells. The UPR^mt^ activates transcription of genes related to glycolysis, mitochondrial biogenesis and translation, as well as heat shock proteins, while suppressing the expression of antimicrobial peptides. Eventually, hemocyte specific OXPHOS perturbation leads to an increase in circulating hemocyte count and hemocyte activation. On an organismal level, this activation of cell-mediated immunity causes the formation of melanotic nodules in uninfected larvae and improves the encapsulation of parasitoid wasps. This beneficial effect is tissue-specific, since knocking down OXPHOS complex genes in the fat body negatively affects development and viability of the hosts and leads to reduced parasitoid wasp encapsulation. Figure created with BioRender.com

Although generally harmful, mitochondrial dysfunction has variable effects depending not only on the severity of the underlying defect but also on the context and the affected tissue-type [47]. We studied the impact of immune tissue-targeted mitochondrial perturbation on the cell-mediated innate immunity in *Drosophila*, which is a powerful in vivo model of immunity and mitochondrial genetics [10]. We targeted the oxidative phosphorylation system, a core mitochondrial function, and knocked down genes from each of the OXPHOS complexes in the main immune tissues of *Drosophila*, the fat body and the hemocytes. The OXPHOS knockdown resulted in antagonistic effects on the immune competence of the host; fat body-targeted mitochondrial perturbations increased susceptibility, while hemocyte-targeted perturbations enhanced the immunocompetence (summary of the main findings presented in Fig. 6). In *Drosophila*, systemically impaired OXPHOS causes low fecundity, poor embryonic survival and a high mortality rate upon bacterial infection [15, 48]. Conversely, it has been indicated that tissue-specific and/or mild mitochondrial dysfunction can be beneficial in specific circumstances. For instance, a reduction in mitochondrial electron transport chain (ETC) function in mammalian muscle cells can protect against apoptosis caused by the cholesterol drug atorvastatin [49]. In the *Drosophila* model, muscle-specific disruption of the ETC slows down age-related muscle deterioration and prolongs lifespan [50]. Importantly, a recent study showed that treating mice with doxycycline, an antimicrobial drug that systemically affects the function of mitoribosomes, and therefore disrupts mitochondrial translation, leads to an increased tolerance against *Escherichia coli* infection without affecting the pathogen load [51].

Knockdown of the OXPHOS complex genes in larval hemocytes resulted in formation of melanotic nodules. These nodules are formed via aberrant activation of the hemocytes and could thus be considered a type of autoimmune response [32, 52–54]. A link between mitochondrial function and melanotic nodules was previously shown in a *Drosophila* cytoplasmic hybrid, aka. cybrid, model [31]. In this model, a mtDNA variant called mtKSA2 containing a cIII gene *mt*:*cyt-b* mutation was shown to induce nodules in multiple nuclear backgrounds [31]. Pharmacological inhibition of cIII with antimycin A mimicked the mtKSA2 nodule phenotype [31]. Here, we show that the nodule phenotype is not cIII-specific, as knocking down nuclear encoded cI, cIV or cV genes in hemocytes also led to the formation of melanotic nodules. Silencing of several mitochondrial ribosomal genes in hemocytes has been found to result in melanotic nodules in *Drosophila* larvae [52]. In addition, larvae fed with the antimicrobial drug ciprofloxacin were shown to accumulate melanized masses [55]. Ciprofloxacin interferes with mitochondrial DNA replication and reduces mtDNA copy number [56]. Based on these data, it is reasonable to surmise that disrupting mitochondrial function, either directly by targeting OXPHOS, or by otherwise reducing mitochondrial performance, is connected with the formation of melanotic nodules, and hence the immune activation of hemocytes.

In addition to melanotic nodules, we observed an enhanced immunocompetence against parasitoid wasps upon OXPHOS gene silencing, together with a lower mitochondrial membrane potential (ΔΨm) in hemocytes. ΔΨm is the electronic charge difference between the intermembrane space and the mitochondrial matrix, mainly generated by the action of the proton pumps functioning in OXPHOS cI, cIII and cIV. Surprisingly, knockdown of cV also caused depolarization of the mitochondrial membrane. Bornhövd et al. [57] showed that destabilizing cV led to a loss of ΔΨm, possibly by altering the fluidity of the mitochondrial inner membrane. Of note, knockdown of OXPHOS cII did not affect ΔΨm and cI knockdown had only a small, non-significant, effect on ΔΨm. In addition to depolarization of the mitochondrial membrane, knocking down cI, III, IV or V resulted in hemocyte profiles mimicking those of wasp-infected larvae, including the formation of immune-activated hemocyte types such as lamellocytes. Interestingly, cI and cII knockdowns, had no effect on ΔΨm or melanotic nodule formation, only mildly affected hemocyte profiles, but improved the encapsulation response against wasps. This suggests that the OXPHOS silencing improves the cell-mediated immune response even without affecting the ΔΨm, but that the loss of ΔΨm is a crucial component in the full manifestation of the immune-enhancing phenotypes we observed.

To better understand how OXPHOS perturbation is linked to the immune-related phenotypes, we conducted a transcriptomic analysis. ΔΨm reduction is a major trigger of stress responses and mitochondrial stress can elicit a mitochondrial unfolded protein response (UPR^mt^) targeted to repair damaged mitochondria. In the UPR^mt^, mitochondrial proteostatic stress leads to a transcriptional response from the nuclear genome, expression of the mitochondrial chaperones and proteases [58], and induces metabolic changes such as increased glycolytic activity [59]. In our transcriptome data, many genes known to be induced by the UPR^mt^, such as heat shock proteins *Hsp60A* and *Hsc70-5* [50, 60, 61], glutathione-S-transferases and genes involved in glycolysis, translation, heme biosynthesis and OXPHOS assembly, were upregulated when *UQCR-C1* was knocked down. In addition, a set of chaperone genes were specifically upregulated at the early timepoint, whereas mitochondrial translation and transport genes were upregulated upon infection in *UQCR-C1* knockdown samples. This transcriptomic response is characteristic of the UPR^mt^ [36, 37]. In addition to loss of ΔΨm and incorrect folding and import of mitochondrial proteins [37, 50, 62], the UPR^mt^ can be induced by OXPHOS dysfunction and mtDNA instability [50, 60]. The decrease in ΔΨm that is caused by the OXPHOS disruption in our model is likely triggering the UPR^mt^ in hemocytes. Interestingly, UPR^mt^ activation has been linked, for example, to drug-induced lifespan extension [63, 64]. By contrast, in our results the hemocyte-specific UPR^mt^ had either no effect on the lifespan (*ATPsynCF6* knockdown females and males, *UQCR-C1* knockdown in males), or significantly reduced it (*UQCR-C1* knockdown females).

Faulty function of the ETC is known to increase ROS, and it was shown earlier that *Drosophila* ROS primes hematopoietic stem cells in the larval hematopoietic organ, the lymph gland, to differentiate into mature hemocytes (crystal cells, plasmatocytes, lamellocytes) [65]. Furthermore, H_2_O_2_ production in hemocytes has been shown to be required for plasmatocyte activation upon bacterial infection and at wound sites [66, 67]. Therefore, we investigated whether ROS plays a role in the hemocyte phenotype produced by the OXPHOS gene knockdowns. We did not observe consistent changes in the expression of ROS scavenging or ROS producing enzymes upon *UQCR-C1* knockdown, nor did we observe changes in overall ROS levels in hemocytes upon knockdown of OXPHOS complexes. Also, silencing antioxidant genes in hemocytes or feeding the antioxidant NAC to the larvae did not affect hemocyte differentiation, demonstrating that ROS production may not be the driving force behind the hemocyte activation resulting from OXPHOS gene knockdown. Of note, *Prx2/Jafrac1*, upregulated by *UQCR-C1* knockdown, has been linked to p38 (a mitogen-activated protein kinase) signaling pathway activation via redox signaling events [68]. p38 signaling, in turn, was shown to induce the expression of one of the three JAK/STAT receptor Domeless ligands (*Upd3*), leading to the activation of JAK/STAT signaling and hypertrophy in the hematopoietic organ, the lymph gland [69]. As JAK/STAT signaling is one of the signaling pathways leading to lamellocyte formation when activated in hemocytes [33] the p38-JAK/STAT axis may also play a role in the hemocytes activation phenotype reported here.

It is also possible that a metabolic switch involving a reduction in mitochondrial OXPHOS activity and an increase in cytosolic aerobic glycolysis in plasmatocytes is a trigger for their activation and transdifferentiation into lamellocytes. Metabolic reprogramming is a key event in mammalian immune cell activation, differentiation and function [70]. Tiwari et al. [71] showed that in the *Drosophila* lymph gland, mitochondrial β-oxidation in progenitor cells is required for hemocyte differentiation, demonstrating that changes in metabolism control cell identity also in the fly. Metabolic reprogramming has also been shown in macrophage activation, where pro-inflammatory macrophages increase aerobic glycolysis and decrease mitochondrial respiration as soon as four hours post exposure to a bacterial-derived lipopolysaccharide [72]. Similarly, *Drosophila* plasmatocytes switch to aerobic glycolysis when activated upon encountering bacteria [46]. Based on our data, knocking down *sima* or *Ldh* is not sufficient to block lamellocyte differentiation and immune activation induced by OXPHOS disruption. However, the RNAi efficiencies of the knockdown lines under control conditions (not infected) were difficult to determine, as the basal levels of gene expression of both genes were low to begin with. Furthermore, hemocyte-targeted cI, cII or cV disruption did not cause consistent changes in the storage of glycogen, increases in the levels of trehalose in the hemolymph or increases in *sima* expression. Of note, *Ldh* expression was upregulated in cIII and cV knockdown hemocytes. Similarly to the knockdown of OXPHOS genes, *Ldh* knockdown in hemocytes led to enhanced, not decreased, encapsulation of wasps. Hence, a metabolic switch from OXPHOS to glycolysis does not seem to be the main driver of the enhanced immunocompetence and hemocyte activation observed in the OXPHOS knockdown larvae.

In *C. elegans*, the UPR^mt^ affects the expression of both glycolytic and innate immune response genes [73]. It is likely that in our model the hemocyte activation and differentiation are induced by a UPR^mt^. We observed the downregulation of many, mostly humoral, innate immune response genes upon *UQCR-C1* knockdown. This is in contradiction with the *C. elegans* model, where the UPR^mt^ caused by mutations in the OXPHOS complexes enhanced immune responses through transcriptional activation of innate immune response genes [37, 73, 74]. These differences could be related to differences in the specific organisation of the immune system in these two species, or systemic vs. tissue-specific OXPHOS perturbations. *C. elegans* is a multicellular organism that lacks many classical attributes of innate immunity such as effector immune cells or Nf-κB signaling [75], while *D. melanogaster* has both humoral and cell-mediated immunity, as well as canonical Nf-κB pathways. The downregulation of genes involved in the humoral innate immune response in our study system could be a mechanism for maintaining the balance between immune responses mediated by the two arms of the innate immune system, and for avoiding harmful inflammation caused by an overactive immune system. In line with this, Campos et al. [73] also reported that a small subset of immune genes was downregulated by the UPR^mt^. The fact that this connection between the UPR^mt^ and innate immunity exists in organisms with drastically different immune system organisation suggests that it is likely a conserved mechanism. In *Drosophila*, FoxO-mediated upregulation of immune response genes as part of the UPR^mt^ response was reported in a study where the cI gene *ND-75* was transiently disrupted in the whole fly [60]. Additional studies are required to elucidate the hemocyte-specific signaling pathway(s) involved in the UPR^mt^ activation.

## Conclusions

We show that mitochondrial function and mitochondrial stress responses clearly play an important role in innate immunity. Several factors influence the effect of mitochondrial perturbation on the host immunocompetence. Ubiquitous or fat body specific knockdown of OXPHOS genes had detrimental effects on viability, the latter impairing the flies’ ability to encapsulate and kill wasps. In contrast, immune cell-targeted OXPHOS perturbations enhanced the cell-mediated immunocompetence of the host. These immune-beneficial effects likely arise from a pre-activation of the immune cells by the UPR^mt^ stress response. The intricate study of the role of the UPR^mt^ in hemocytes is complicated by the difficulty of experimentally inducing it without directly inducing changes in other processes such as ROS production and metabolism. However, we now show for the first time that tampering with the mitochondrial function in hemocytes launches an UPR^mt^-like transcriptomic profile including upregulation of genes involved in glycolysis, heat shock response and mitochondrial translation, leading to immune activation of hemocytes.

## Methods

### Targeted gene silencing

The binary GAL4/UAS expression system [76] was utilized for targeted gene silencing using RNA interference (RNAi). To express the UAS-RNAi constructs ubiquitously, the *da-GAL4* (Bloomington Drosophila Stock Center, Indiana University, Bloomington, BL #8641) driver was used. For fat body-specific gene silencing, the *Fb-GAL4* driver was used [77, 78]. For silencing the gene expression in hemocytes, a combination of two hemocyte GAL4 drivers, *Hml*^*Δ*^*-GAL4* (BL #30,139, [79]) and *He-GAL4.Z* (BL #8699, [33]), was used and referred to as *Hml*^*Δ*^ > *;He* > *.* Of note, *He-GAL4* is expressed in all hemocyte classes, including lamellocytes whereas *Hml*^*Δ*^*-GAL4* is not expressed in lamellocytes [33, 79]. RNAi strains and their respective genetic background *w*^*1118*^ strains (called hereafter *w*^*GD*^ and *w*^*KK*^) were obtained from the Vienna Drosophila Resource Center (VDRC). The GAL4 drivers and UAS-RNAi strains are listed in Additional file 1: Table S1 and Table S2. GAL4/UAS crosses were performed with 15 GAL4 virgin females and 7 UAS-RNAi males.

### Fluorescent hemocyte reporter strains and overexpression lines

To identify hemocyte types, the in vivo hemocyte reporters the *eater-GFP* (plasmatocytes, [80]) and *msnF9mo-mCherry* (lamellocytes, [81]) combined with *Hml*^*Δ*^*-GAL4; He-GAL4* [28] were utilized. The combination strain is referred to as *Me; Hml*^*Δ *^>; *He* > *. Toll*^*10b*^ and *hep*^*CA*^ overexpression lines [33] were a kind gift from Professor Dan Hultmark.

### Fly maintenance

Flies were reared on a diet containing 36 g of mashed potato powder, 9 g of agar, 45.5 ml corn syrup, 14.5 g of dry yeast, 8 g of nipagin and 5 g of ascorbic acid per 1 L of water. Fly strains were maintained at 25 °C ± 0.5 °C. Experimental crosses were kept at a 12:12LD cycle at 25 °C ± 0.5 °C for the first egg laying day and vials with eggs were either kept at 25 °C ± 0.5 °C or moved to 29 °C ± 0.5 °C (60% humidity), depending on the experiment.

### Viability assay

Viability was assayed from ubiquitous, fat body or hemocyte-targeted OXPHOS complex I-V gene knockdowns. Females of the parental crosses were left to lay eggs at 25 °C for two subsequent days, and the egg vials were transferred to 29 °C approximately 1 day after the start of the egg lay. The developmental stage of the larvae was checked on the fifth and sixth day after the start of the egg lay. A final check was done on the eight day, when control flies had already eclosed.

### Hemocyte RNA isolation

3rd instar larvae were dissected in ice cold 1 × Phosphate Buffered Saline (PBS) on a 12-well glass slide to release the hemolymph. The hemolymph from 50 larvae (males and females separately) was pooled per sample (three replicate samples per genotype) and centrifuged for 7 min at 2500 g at 4 °C to pellet the hemocytes. As much of the PBS was removed as possible (the hemocyte pellet is not visible and is loosely attached on the Eppendorf tube) prior to storing the samples at -80 °C. Total RNA was extracted with the Single Cell RNA Purification Kit (Norgen Biotek) protocol that is optimized for extracting RNA from low numbers of cells. Samples were treated with DNase I (RNAse free DNase I kit, Norgen Biotek) as described in the manufacturer’s protocol. The RNA quality was checked using a NanoDrop ND-1000 spectrophotometer (Thermo Scientific) and each sample was diluted to 10 ng/µl prior to storing at -80 °C.

### Mitochondrial membrane potential

The MitoProbe™ tetramethylrhodamine methyl ester (TMRM) Assay Kit for Flow Cytometry (Invitrogen) was used to detect changes in the mitochondrial membrane potential (ΔΨm) in hemocytes in response to the knockdown of genes of the OXPHOS complexes. Hemocytes from 20 male 3rd instar larvae per sample were used for the analysis. The larvae were washed and then dissected in 1 × PBS. Samples were stained with 0.4 nM TMRM for 30 min, protected from light. The negative control sample was treated with 1 µM carbonyl cyanide m-chlorophenylhydrazone (CCCP) for 5 min prior to the staining in order to depolarize the mitochondrial membrane. The samples were then analyzed using a CytoFlex S flow cytometer (Beckman Coulter). The following channels and gain settings (in parentheses) were used: FSC (20), SSC (40), FITC (to detect GFP, 40), PE (TMRM, 80) and EDC (*mCherry*, 45). Non-fluorescent hemocytes, and hemocytes expressing/stained with each fluorophore individually were used to calculate the color compensation values and to determine the level of autofluorescence of the hemocytes. Hemocytes were classified as described above, and eaterGFP^high^ plasmatocytes were used in the analyses to control for the hemocyte type.

### RT-qPCR

RT-qPCR was used to quantify the efficiency of the knockdown of OXPHOS genes, *Ldh* and *sima*, and to detect the expression levels of the genes of interest in the larval hemocytes. For the RT-qPCR reactions, the iTaq Universal Sybr green One-step kit (Bio-Rad) was used. 10 µl reaction mix per sample was as follows: 5 µl of iTaq universal SYBR Green reaction mix (2x), 0.125 µl of iScript reverse transcriptase (RT), 0.3 µl of forward and reverse primers each (300 nM each), 2 µl of template RNA (20 ng per reaction) and 2.275 µl of nuclease free H_2_O. Each run included a sample without RT and a sample without an RNA template for quality checking. The samples were run with a Bio-Rad CFX96 Real-time PCR system with a reverse transcription reaction for 10 min at 50 °C, polymerase activation and DNA denaturation for 1 min at 95 °C followed by 39 cycles of amplification (denaturation for 10 s at 95 °C, annealing/extension for 15 s at 60 °C) and a melt-curve analysis at 65–95 °C in 0.5 °C increments. The efficiency of each primer pair was calculated, and a primer pair was deemed usable, if the efficiency fell between 90–110% (Additional file 1: Table S4). The efficiencies were calculated based on the slope of the regression line obtained from a fivefold serial dilution of the template RNA (each point assayed in duplicate), using the following formula: Efficiency (%) = (-1/10^slope^ – 1) × 100 built into the Bio-Rad software. Primers, primer sequences and their efficiencies are shown in Table S4. The average threshold cycle (Ct) values were calculated from two technical replicates per three biological replicates. In all analyses, *Histone H3.3B* (*His3.3B*) was used as a reference gene.

### Lifespan assay

For the lifespan assay, 0–2 day old virgin females and males were kept in standard food vials in pools of 10 flies. 10 biological replicates were included from both sexes. Flies were flipped three times per week, with the number of dead flies was recorded simultaneously.

### Melanotic nodules

3rd instar larvae were individually dissected in a drop of water, and the presence of melanotic nodules was determined using a stereomicroscope. 100 larvae per genotype were checked for the presence of melanotic nodules. Examples of larvae and flies exhibiting melanotic nodules (20 × magnification), and dissected melanotic nodules (40 × magnification), were imaged using a Deltapix Invenio 10EIII camera (DeltaPix, Denmark) and Nikon SMZ745T stereomicroscope using the DeltaPix InSight software.

### Parasitoid wasp assay

The parasitoid wasp *Leptopilina boulardi* strain G486 was used as a natural model to induce the cell-mediated innate immune response in *Drosophila* larvae. Female flies were allowed to lay eggs for 24 h at 25 °C. The eggs were then transferred to 29 °C for further development. 2nd instar larvae (~ 72 h after egg lay) were exposed to 12–15 female wasps for two hours at 22 °C, after which the wasps were removed, and the larvae placed back at their rearing temperature. The encapsulation response of the *Drosophila* larvae was checked from 3rd instar larvae 48 h post infection, by dissecting the larvae individually in a drop of water under a stereomicroscope. The cellular immune response of the larvae was determined based on their ability to fully encapsulate and melanize the wasp egg/larva. Larvae containing either partially melanized or completely non-melanized wasp egg/larvae were deemed as having failed in their immune response. The fly larvae containing more than two non-melanized wasp eggs or larvae were excluded from the experiment. Three replicate crosses per genotype were made, and larvae were dissected until 50 infected larvae per replicate had been collected (150 larvae per genotype in total).

### Fluorescent hemocyte reporter analysis

Larvae were reared at 29 °C until the 3rd instar stage was reached, washed 3 times with filtered water and individually dissected in 20 µl of 1% Bovine Serum Albumin (BSA) in 1 × PBS. Each sample was topped up to 100 µl with 1% BSA in PBS and analyzed using an Accuri C6 flow cytometer (Becton Dickinson). Each control and OXPHOS RNAi strain was tested on three separate occasions. Hemocytes were classified based on the fluorescence intensity of the *eater-GFP* and *msn-mCherry* reporters. One-color controls (non-fluorescent, GFP-only and *mCherry*-only hemocytes) were used to set up the gates and to correct fluorescence spill-over to the wrong channel, as described in [28]. GFP was detected using a 510/20 nm filter and mCherry using a 610/20 nm filter, excitation was with the 488 nm laser. With this method, five hemocyte populations can be detected, two of which express highly the plasmatocyte reporter *eater-GFP* (GFP^high^; plasmatocytes and GFP^high^mCherry^low^; activated plasmatocytes), the rest having reduced *eater-GFP,* along with an increase in *mCherry* expression (GFP^low^; lamelloblasts, GFP^low^mCherry^low^; prelamellocytes and mCherry^high^; lamellocytes). 

### RNA sequencing

Hemocyte samples were collected and RNA was extracted for the RNA sequencing as described above. RNA sequencing samples contained hemocytes from 100 3rd instar male larvae, collected in triplicate. Female samples were collected simultaneously for RT-qPCR-based gene expression measurements. The next-generation sequencing was conducted at the Finnish Functional Genomics Center (Turku, Finland). Briefly, sample quality was ensured using Advanced Analytical Fragment Analyzer and sample concentration was measured with Qubit® (Life Technologies). Library preparation was done by using the Illumina Stranded mRNA Library Preparation kit, with 100 ng of total RNA as starting material. The sequencing run was performed using Illumina NovaSeq 6000 and base calling was done with bcl2fastq2 conversion software (NovaSeq 6000).

### RNA sequencing data analysis

The sequenced RNA reads were aligned to *D. melanogaster* reference genome dm6 using STAR aligner version "2.7.10a". NCBI Reference Sequence Database gene model table was used for performing gene transcript annotation. The R packages edgeR [82] and limma [83] were used for the differential gene expression analyses. The data were normalized and transformed to CPM for making the comparisons between sample groups. The data were analyzed using linear models. Genes that had no detectable expression in all sequenced samples and genes where fold change in comparison to control was less than 1.5 were excluded from the analyses. False discovery rate (FDR) less than 0.1 was considered significant.

### ROS detection

Male UAS-RNAi flies were crossed with females with a version of the hemocyte driver without the *UAS-GFP* constructs (*w*^*1118*^*;* + *;Hml*^*Δ*^*-GAL4,He-GAL4;* Additional file 1: Table S2) and the progeny were reared at 29 °C. 3rd instar male larvae were washed 3 times with filtered water and hemolymph from pools of 20 larvae were bled in 250 µl of 1 × Schneider’s Drosophila Medium (Gibco) on ice to obtain hemocytes. 500 nM of the CellROX™ green reagent (molecular probes for Life Technologies) diluted in Dimethyl Sulfoxide (DMSO) was added to 250 µl of sample to assess the level of oxidative stress in hemocytes. The CellROX™ is a probe-based system, where the probe is only weakly fluorescent in the reduced state and becomes strongly fluorescent under oxidative conditions. After 1-h incubation at room temperature in the dark, 2.5 µl of Propidium iodide (PI) was added and the samples were immediately analyzed using a CytoFlex S flow cytometer with the following settings: FSC (20), SSC (40), FITC (40) and PC5.5 (PI, 170). As a negative control, larvae were fed with 1 mg/ml NAC starting from the egg stage.

### NADH quantification

Lactate dehydrogenase activity assay kit (Sigma-Aldrich) was used to detect the reduction of NAD to NADH by Ldh. Hemocytes from pools of 80–200 mid to late 3rd instar larvae (sexes pooled; three replicates per a genotype) were collected by dissecting the larvae to release the hemolymph in 1 × PBS on ice. Samples were centrifuged for 7 min at 2500 g at 4 °C to pellet the hemocytes and stored at -80 °C until the analysis. An aliquot of each sample was taken for measuring the total protein concentration using a BCA assay kit (Merck Millipore). The analysis was conducted following the manufacturer’s protocol, except for scaling down the initial amount of the LDH assay buffer (from 500 µl to 200 µl) and incubating the samples at 23 °C instead of the instructed 37 °C optimized for mammalian cells. The absorbance was measured at 450 nm with a Wallac Envision 2104 Multilabel Reader (PerkinElmer) and the readings at the start and at the end (66 min after the initial reading) were recorded. A NADH standard curve and a positive control were provided in the kit and run in duplicate, as were the experimental samples. Three concentrations of the hemocyte samples were each run in duplicate (1:25; 2:5; and non-diluted). Due to the sample concentrations in the two dilutions being too low, the non-diluted samples were used in the analysis. The difference between the end and the start was calculated and used to determine the amount of NADH generated, based on the standard curve.

### Glycogen and trehalose measurements

Glycogen, glucose and trehalose were measured using the GAGO-20 kit (Sigma Glucose (GO) assay kit) following the protocol in [84]. For glycogen, pools of seven mid-to-late 3rd instar larvae per sample were homogenized in 100 µl of 1 × PBS. An aliquot of the sample was taken for measuring the total protein concentration using a BCA assay kit (Merck Millipore). The remaining sample was heat-treated (70 °C, 10 min) and centrifuged at 14 000 g at 4 °C for 3 min. The supernatant was diluted to 1:5 and pipetted into a 96-well plate in duplicate. The assay was performed according to the GAGO-20 kit protocol. One duplicate was treated with GO reagent only and the other with GO reagent and amyloglucosidase (1 µl of enzyme per 1 ml of GO reagent) to break down glycogen into glucose. A blank (30 µl of 1 × PBS), a glucose standard (twofold serial dilutions from 0.01 to 0.16 mg/ml glucose) and a glycogen standard (twofold serial dilutions from 0.01 to 0.16 mg/ml glycogen) treated with amyloglucosidase were also included. The samples were incubated for 30 min at 37 °C, and 100 µl of 12 N sulfuric acid (H2SO4) was added to stop the enzymatic reaction. The absorbance at 540 nm was measured with a Wallac Envision 2104 Multilabel Reader (PerkinElmer). The amount of free glucose was determined from untreated samples using the glucose standard curve. To calculate the amount of glycogen, the absorbance measured for free glucose in the untreated samples was subtracted from the absorbance of the samples treated with amyloglucosidase. The amount of glycogen was then determined from the glycogen standard curve.

For determining trehalose content, pools of 20 larvae were washed carefully in filtered water. Larvae were pricked using stainless steel Austerlitz insect pins of 0.2 mm diameter (Entomoravia), horizontally from the posterior end towards the anterior end, avoiding damaging the gut. Pricked larvae were collected in a 100 µm pluriStrainer mini 100 filter placed in a 1.5 ml Eppendorf tube on ice. The samples were centrifuged for 7 min at 5000 g at 4 °C and 1 µl of hemolymph was collected. 1 µl of hemolymph was then mixed with 99 µl of trehalase buffer (TB, 5 mM Tris pH 6.6, 137 mM NaCl, 2.7 mM KCl). 25 µl aliquots of samples were snap-frozen and stored for a protein concentration measurement. After heat-inactivating the samples (70 °C, 5 min), each sample was divided in two aliquots, and 3 µl of Porcine kidney trehalase (Sigma) was added to one of the aliquots to break down trehalose into glucose. After an overnight incubation with the enzyme, 30 µl of a blank (1 × PBS), a glucose standard (twofold serial dilutions from 0.01 to 0.16 mg/ml glucose) and duplicates of the samples were pipetted into a 96-well plate and the assay was then performed according to the GAGO-20 kit protocol as described above. The absorbance at 540 nm was measured with a Wallac Envision 2104 Multilabel Reader. The amount of free glucose in the untreated samples was determined with the help of the glucose standard curve. The amount of trehalose was calculated by first subtracting the absorbance of free glucose from the samples treated with trehalase, and then using the trehalose standard curve to calculate the trehalose concentration.

### Statistical analyses

R version 4.0.4 and Excel version 16.60 (Microsoft) were used for analyzing the data and R was used for plotting. The data on proportions (wasp assay, ROS-positive cells) were analyzed using logistic regression with a binomial distribution with the lm4 package [85]. In the case of the immune response against wasps, non-melanized and partially melanized cases were deemed as a failure, and fully melanized as a success. When needed, pairwise comparisons of the strains were analyzed using the multcomp package [86]. The data on cell counts were analyzed using a generalized linear model with negative binomial distribution using the MASS package [87]. The least square means (estimated marginal means) were analyzed for multiple comparisons among the strains and the Tukey method was used for adjusting the p-value using the emmeans package [88]. Data on trehalose and glycogen concentrations and gene expression were analyzed using Kruskall-Wallis tests followed by a pairwise student’s t-tests or the Wilcox tests. Data on mitochondrial membrane potential were analysed using a one-sample t-test. The lifespan assay data were analyzed using R packages ggsurvfit and survminer [89, 90], and a log-rank test for comparing survival between groups.

### Supplementary Information

**Additional file 1: Table S1.** RNAi strains. **Table S2.** Drivers and reporters. **Table S3.** Developmental time and viability of OXPHOS KD flies. **Table S4.** RT-qPCR primers.

**Additional file 2: Fig. S1.** Hemocyte-targeted OXPHOS RNAi constructs efficiently silence the OXPHOS gene expression and alter the mitochondrial membrane potential in hemocytes. (A-A’) Knockdown efficiencies of OXPHOS complex I (*ND-75*), cII (*SdhD*), cIII (*UQCR-C1*), cIV (*COX5B*) and cV (*ATPsynCF6*) RNAi constructs from the VDRC GD library (STable 1) in (A) female and (A’) male larval hemocytes. cIII gene *ox* and cIV gene *COX5B* knockdown efficiencies could not be tested due to the short gene length largely occupied by the RNAi hairpin structure. The percentages refer to the mean reduction in the mRNA levels in the OXPHOS knockdowns compared to those in the control hemocyte samples. (B) Histograms showing mitochondrial membrane potential in plasmatocytes measured by flow cytometry using the MitoProbe™ TMRM stain.

**Additional file 3: Fig. S2.** Melanotic nodule prevalence and a detailed hemocyte analysis of OXPHOS complex I-V knockdown in RNAi strains from the KK library. (A) Quantification of melanotic nodules detected in the larvae (*n* = 100). (B-B’) Survival curves of female (B) and male (B’) *UQCR-C1* and *ATPsynCF6* knockdown flies and controls maintained at 25 °C. Log-rank test was used to compare differences in survival between groups. *** *p* < 0.001, *n* = 82–100. (C–C’’’’’) Quantification of total hemocytes and classification of hemocyte types based on *eater-GFP* and *msn-mCherry* expression when knocking down selected OXPHOS complex I-V genes in hemocytes (*n* = 30). Complex-specific target genes are: cI—*ND-75*, cII—*SdhD*, cIII—*UQCR-C1*, cIV—*COX5B*, cV—*ATPsynCF6*. (C–C’’) Total, plasmatocyte (pc) and activated plasmatocyte (act pc) counts. (C’’’-C’’’’’) Lamelloblast (lb), prelamellocyte (pre lc) and lamellocyte (lc) counts. The data were analyzed using a generalized linear model with a negative binomial distribution. Stars indicate the statistical difference of the OXPHOS gene knockdowns compared to the KK library background control (*w*^*KK*^). ns = not significant, * *p* < 0.05, ** *p* < 0.01, *** *p* < 0.001.

**Additional file 4: Fig. S3.** Encapsulation response in RNAi strains from the KK library and hemocyte composition of selected GD library strains after infection. (A) Encapsulation efficiency assayed 48 h after *L. boulardi* infection in controls and after OXPHOS gene silencing in hemocytes using the RNAi strains from the VDRC KK library. (B-B’’’’’) Quantification of total hemocytes and classification of hemocyte types based on *eater-GFP* and *msn-Cherry* expression when knocking down selected OXPHOS genes in hemocytes (*n* = 30) and infecting the larvae with *L. boulardi* wasps. Complex-specific target genes from the GD library: cI—*D-75*, cIII—*UQCR-C1* and cV—*ATPsynCF6*. (B-B’’) Total, plasmatocyte (pc) and activated plasmatocyte (act pc) counts. (B’’’-B’’’’’) Lamelloblast (lb), prelamellocyte (pre lc) and lamellocyte (lc) counts. The data were analyzed using a generalized linear model with a negative binomial distribution. Stars indicate the statistical difference of the OXPHOS gene knockdowns to the background control. ns = not significant, * *p* < 0.05, ** *p* < 0.01, *** *p* < 0.001.

**Additional file 5: Fig. S4.** Hemocyte analysis of control and *UQCR-C1* hemocyte knockdown larvae 90, 92 and 96 h after egg lay. (A-A’’) Total, plasmatocyte (pc) and activated plasmatocyte (act pc) counts. (A’’’-A’’’’’) Lamelloblast (lb), prelamellocyte (pre lc) and lamellocyte (lc) counts. The data were analyzed using a generalized linear model with a negative binomial distribution. (B-B’) Mitochondrial membrane potential in control and in *UQCR-C1* knockdown hemocytes 90 h after egg lay was measured using the MitoProbe™ TMRM stain. The data were analyzed using one-sample t-test, comparing the ratio to a value of 1. ns = not significant, * *p* < 0.05, ** *p* < 0.01, *** *p* < 0.001.

**Additional file 6. ** Differentially expressed genes, their log2 fold changes, *p*-values and FDR corrected *p*-values per comparison.

**Additional file 7. ** Lists of upregulated lamellocyte marker genes**.**

**Additional file 8. ** Significant GO terms per comparison.

**Additional file 9: Fig. S5.** ROS production is not crucial for the OXPHOS perturbation-related enhancement in immune response. (A) Heatmap showing the gene expression changes of genes related to reactive oxygen species (ROS) detoxification in early and late timepoints after *UQCR-C1* knockdown and after wasp infection. (B-B’) Reactive oxygen species (ROS) were measured using the CellROX™ Green reagent. (B) CellROX™ Green signal in control hemocytes and in hemocytes obtained from N-acetyl cysteine (NAC)-fed larvae. (B’) CellROX™ Green signal in control hemocytes and in hemocytes with a knockdown of OXPHOS cI, cIII or cV genes. (C–C’) ROS levels were quantified as (C) a ratio of the CellROX™ Green signal in the OXPHOS complex knockdown hemocytes to that of the control hemocytes and as (C’) a proportion of hemocytes positive for the CellROX™ Green signal (*n* = 3, 5000–7000 hemocytes per replicate). cI = *ND-75*, cIII = *ox*, cV = *ATPsynCF6*. The ratios were analyzed using one-sample t-tests, comparing the ratios to a value of 1. The percentages were analyzed using a logistic regression with a binomial distribution. ns = not significant, * *p* < 0.05, ** *p* < 0.01, *** *p* < 0.001. (D) Control and antioxidant (AO) gene knockdown larvae were infected with *L. boulardi* wasps and the melanization response against the wasp eggs and larvae was assessed (*n* = 150). The data were analyzed using logistic regression with a binomial distribution. Replication was included as a random factor in the analyzes. (E-E’’’’’) Quantification of total hemocytes and hemocyte types classified based on *eater-GFP* and *msn-Cherry* expression when knocking down the AO genes in hemocytes of male larvae (*n* = 10). Of note, control samples are the same as in Fig. 2 C–C’’’’’ (*n* = 30). Asterisks indicate the statistical difference between the AO knockdowns and the control. Error bars indicate standard error of the mean. (F-F’’’’) Hemocyte quantification from control (*w*^*GD*^) and *UQCR-C1* knockdown male larvae fed with normal food (-) or food containing 1 mg/ml NAC ( +), (*n* = 30 for *w*^*GD*^-; 40 for *UQCR-C1*^*GD*^-; 35 for *w*^*GD*^ + and 39 for *UQCR-C1*^*GD*^ +). The data on hemocyte counts were analyzed using a generalized linear model with a negative binomial distribution. ns = not significant, * *p* < 0.05, ** *p* < 0.01, *** *p* < 0.001.

**Additional file 10: Fig. S6.** Knocking down *Ldh* or *sima* in hemocytes does not affect hemocyte activation or differentiation. (A-B’) Expression of (A-A’) *sima* and (B-B’) *Ldh* was measured in hemocytes of 3rd instar OXPHOS knockdown (GD library) female and male larvae and is shown here normalized to the expression of the control gene *His3.3B* expression, and relative to the gene expression level in the hemocytes of control animals. cI—*D-75*, cII – *SdhD*, cIII—*ox* and cV—*ATPsynCF6*. Data on overall differences were analyzed using the Kruskal–Wallis test. When needed, pairwise differences were analyzed using the t-test, applying Bonferroni method to correct for multiple comparisons. ns = not significant, * *p* < 0.05, ** *p *< 0.01, *** *p* < 0.001. (C–C’) Knockdown efficiencies of *sima* and *Ldh* RNAi constructs in hemocytes of (C) female and (C’) male larvae normalized to *His3.3B* expression. Differences between the knockdowns and controls were analyzed using t-tests. (D) *Ldh* gene expression in control and *sima* knockdown hemocytes in males, normalized to *His3.3B* expression. (E) Production of NADH was measured as a readout of Ldh activity in control and *Ldh* knockdown hemocytes. Data shown is normalized to the amount of protein (µg/µl) to account for different amounts of starting material. Data in (D-E) were analyzed with t-tests. (F-F’’’’’) Quantification of total hemocytes and classification of hemocyte types based on *eater-GFP* and *msn-mCherry* expression in controls, in *UQCR-C1* and *sima* single knockdown, as well as in *sima; UQCR-C1* and *Ldh; ATPsynCF6* double knockdowns (*n* = 30). Data from female larvae are presented. Two backgrounds strains (*w*^*GD*^ and* w*^*KK*^) had very similar hemocyte profiles (similarly to males in Fig. 5C-C’’’’’), but with some statistically significant differences (underlined symbols above them). Not underlined significance symbols above the samples indicate their difference to the control. In the case of the double knockdowns, lower underlined symbol indicates the difference to either *UQCR-C1* or *ATPsynC6* single knockdowns and upper symbol in grey the difference to the control. total = total circulating hemocyte count, pc = plasmatocytes, act pc = activated plasmatocytes, lb = lamelloblasts, pre lc = pre-lamellocytes, lc = lamellocytes. The data were analyzed using a generalized linear model with a negative binomial distribution. ns = not significant, * *p* < 0.05, ** *p* < 0.01, *** *p* < 0.001.

**Additional file 11: Fig. S7.** Knockdown of the OXPHOS genes in hemocytes does not cause major changes in glycogen or trehalose levels. (A-A’) Storage sugar glycogen content normalized to protein content in (A) female and (A’) male larvae with OXPHOS knockdown in hemocytes. (B-B’) Content of circulating trehalose in hemolymph normalized to protein content in (B) female and (B’) male larval hemolymph with OXPHOS knockdown in hemocytes. cI—*D-75*, cIII—*ox* and cV—*ATPsynCF6.* The data were analyzed using pairwise t-tests, comparing each knockdown to the control (*w*^*GD*^). ns = not significant, * *p* < 0.05, ** *p* < 0.01, *** *p* < 0.001.

## Data Availability

The RNA-seq data from this study have been submitted to NCBI Gene Expression Omnibus (GEO) accession number GSE237367 [91, 92].

## Abbreviations

OXPHOS: Oxidative phosphorylation
mtDNA: Mitochondrial genome
ROS: Reactive oxygen species
DAMP: Damage-associated molecular pattern
AMP: Antimicrobial peptide
ΔΨm: Mitochondrial membrane potential
CCCP: Carbonyl cyanide 3- chlorophenylhydrazone
AEL: After egg lay
PCA: Principal component analysis
CPM: Counts per million
GO: Gene Ontology
UPR^mt^: Mitochondrial unfolded protein response
H_2_0_2_: Hydrogen peroxide
NAC: N-acetylcysteine
NADH: Reduced form of nicotinamide adenine dinucleotide
ETC: Electron transport chain
MAPK: Mitogen-activated protein kinase
RNAi: RNA interference
VDRC: Vienna Drosophila Resource Center
TMRM: Tetramethylrhodamine methyl ester
RT-qPCR: Reverse Transcription Quantitative Real-time PCR
RT: Reverse transcriptase
Ct: Threshold cycle
BSA: Bovine Serum Album
PBS: Phosphate Buffered Saline
FDR: False discovery rate
DMSO: Dimethyl Sulfoxide
PI: Propidium iodide
