# Supplementary material for: Mitochondrial perturbation in immune cells enhances cell-mediated innate immunity in Drosophila
Source: BMC Biol. 2024 Mar 13;22:60. doi: 10.1186/s12915-024-01858-5 (PMC10935954; doi:10.1186/s12915-024-01858-5)
Supplement: Supplementary file 1 — Additional file 1: Table S1. RNAi strains. Table S2. Drivers and reporters. Table S3. Developmental time and viability of OXPHOS KD flies. Table S4. RT-qPCR primers. [file 12915_2024_1858_MOESM1_ESM.docx]

**Table S1.** Vienna Drosophila Resource Center (VDRC) RNAi lines used in the experiments. VDRC RNAi line genetic backgrounds are *w^1118^* #6000 for the GD library (*w^GD^*) and #60100 for the KK library (*w^KK^*).

| **Target gene** | **Gene name** (OXPHOS complex association) | **CG ID** | **VDRC ID/ background** | **Inserted chromosome** |
| --- | --- | --- | --- | --- |
| *ND-75* | *NADH dehydrogenase (ubiquinone) 75 kDa subunit* (cI) | CG2286 | #52047/GD | 3 |
| *ND-75* | *NADH dehydrogenase (ubiquinone) 75 kDa subunit* (cI) | CG2286 | #100733/KK | 2 |
| *SdhD* | *Succinate dehydrogenase, subunit D* (cII) | CG10219 | #26776/GD | 2 |
| *SdhD* | *Succinate dehydrogenase, subunit D* (cII) | CG10219 | #101739/KK | 2 |
| *ox* | *oxen* (cIII) | CG8764 | #35829/GD | 2 |
| *UQCR-C1* | *Ubiquinol-cytochrome c reductase core protein 1* (cIII) | CG3731 | #40466/GD | 3 |
| *UQCR-C1* | *Ubiquinol-cytochrome c reductase core protein 1* (cIII) | CG3731 | #101350/KK | 2 |
| *COX5B* | *Cytochrome c oxidase subunit 5B* (cIV) | CG11015 | #30892/GD | 3 |
| *COX5B* | *Cytochrome c oxidase subunit 5B* (cIV) | CG11015 | #105769/KK | 2 |
| *ATPsynCF6* | *ATP synthase, coupling factor 6* (cV) | CG4412 | #35385/ GD | 2 |
| *ATPsynCF6* | *ATP synthase, coupling factor 6* (cV) | CG4412 | #107826 /KK | 2 |
| *sima** | *similar* | CG45051 | #106504 /KK | 2 |
| *Ldh* | *Lactate dehydrogenase* | CG10160 | #31192GD | 3 |
| *Sod1* | *Superoxide dismutase 1* | CG11793 | #31551 /GD | 3 |
| *Sod2* | *Superoxide dismutase 2* | CG8905 | #42162 /GD | 2 |
| *Cat* | *Catalase* | CG6871 | #6283 /GD | 3 |

*also known as *HIF-1α; Hypoxia inducible Factor 1α*

| **Table S2.** Driver and reporter constructs used in the experiments.   \| **Construct name** \| **Genotype** \| **Target tissue** \| \| --- \| --- \| --- \| \| *Hml^Δ^ >;He>* \| *w[1118];Hml[delta]-GAL4,UAS-eGFP;He-GAL4,UAS-GFP* \| Hemocytes, contains GFP \| \| *Hml^Δ^ >,He>* \| *w[1118];+;Hml[delta]-GAL4,He-GAL4* \| Hemocytes, no GFP \| \| *Fb>* \| *w[1118]; Fb-GAL4,UAS-GFP; +* \| Fat body \| \| *Da>* \| *w[1118];+;Da-GAL4,UAS-GFP* \| Ubiquitous \| \| *Me;HH>* \| *yw,eater-GFP,MSNF9mo-mCherry; Hml-GAL4[delta];He-GAL4* \| Hemocytes (+ hemocyte reporters) \| |  |
| --- | --- | --- | --- | --- | --- | --- | --- | --- | --- | --- | --- | --- | --- | --- | --- | --- | --- | --- | --- |

**Table S3.** Tissue specific effects of OXPHOS complex gene knockdowns on development time (egg-to-pupae) and viability (eclosion of adults). OXPHOS complex I-V specific genes (constructs from both GD and KK VDRC RNAi libraries) were knocked down ubiquitously (*da-GAL4>*), and from the main immune tissues; the fat body (*Fb-GAL4>*) and the hemocytes (*Hml^Δ^ -GAL4;He-GAL4>*). Control flies (*da-GAL4*, *FB-GAL4* or *Hml^Δ^ -GAL4;He-GAL4* females crossed with *w^1118^* males) developed at normal rate and eclosed within approximately eight days after egg laying when reared at 29 °C. If the length of the larval or pupal stages or the eclosion of the adults deviated from those of the controls, the OXPHOS gene knockdown was determined to cause a development delay.

| **RNAi strain** | **OXPHOS Complex** | **Whole fly (da-GAL4>)** | **Fat body (Fb-GAL4>)** | **Hemocytes (HH-GAL4>)** |
| --- | --- | --- | --- | --- |
| *UAS-ND-75* GD | cI | Dev.delay, pupal lethal | Dev.delay, viable | viable |
| *UAS-ND-75* KK | cI | Dev.delay, 2^nd^ instar lethal | Dev.delay, viable | viable |
| *UAS-SdhD* GD | cII | Dev.delay, 3^rd^ instar lethal | viable | viable |
| *UAS-SdhD* KK | cII | Dev.delay, 3^rd^ instar lethal | viable | viable |
| *UAS-ox ^29^*GD | cIII | Dev.delay, 2^nd^ instar lethal | Dev.delay, pupal lethal | viable |
| *UAS-UQCR-C1* GD | cIII | Dev.delay, 1^st^ instar lethal | Dev.delay, pupal lethal | viable |
| *UAS-UQCR-C1* KK | cIII | Dev.delay, 1^st^ instar lethal | Dev.delay, pupal lethal | viable |
| *UAS-Cox5B* GD | cIV | Dev.delay, 2^nd^ instar lethal | Dev.delay, pupal lethal | viable |
| *UAS-Cox5B* KK | cIV | Dev.delay, 2^nd^ instar lethal | Dev.delay, pupal semilethal | viable |
| *UAS-ATPSynCF6* GD | cV | Dev.delay, 1^st^ instar lethal | Dev.delay, pupal lethal | viable |
| *UAS-ATPSynCF6* KK | cV | Dev.delay, 2^nd^ instar lethal | Dev.delay, pupal semilethal | viable |

**Table S4**. RT-qPCR primers.

| **Gene** | **Primer sequence 3’🡪 5’** | **Efficiency %** |
| --- | --- | --- |
| *ND-75* | F_TCCGTAAGGTGAGCAGCATC; R_TCAGCAAGCCATTCCTCGTT | 103.4 |
| *SdhD* | F_ ATGTCCCTCTCGTTGCTTCTG; R_ CTTCAGGGGAGTGATGCGAG | 97.3 |
| *UQCR-C1* | F_CGCTCCGAGAACGAGAAGAA; R_AGTGTAGGCGTTCAAGTGGG | 108.9 |
| *ox* | F_ATCATCGCGTCGGCCTTTTT; R_ TGATGCCCTCGAAAATCGCA | 100.5 |
| *ATPsynCF6* | F_CTGGACAAAGTGCGCGAGTA; R_CACGGTCCAGTTCGGTCTTC | 103 |
| *Ldh* | F_ACACCGACATCCTCAAGAACAT; R_CGGGATTGGACACCATAAGCA | 104.1 |
| *sima* | F_CCGTTGGGAACTAGCACCTT; R_CCATGCTGGCACGAAAACAA | 93.7 |
| *His3.3b* | F_CGCTCAGGATTTCAAGACCG; R_GGATGTCCTTAGGCATGATTGT | 102 |
