# Supplementary figures and images for: Mitochondrial perturbation in immune cells enhances cell-mediated innate immunity in Drosophila
Source: BMC Biol. 2024 Mar 13;22:60. doi: 10.1186/s12915-024-01858-5 (PMC10935954; doi:10.1186/s12915-024-01858-5)

A

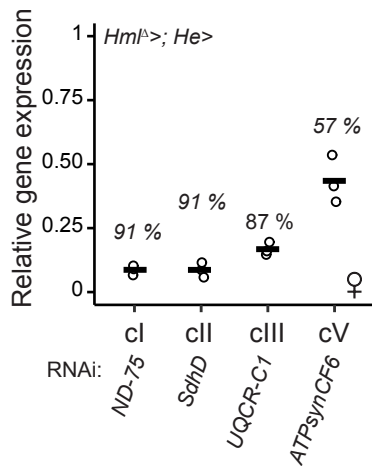

A'

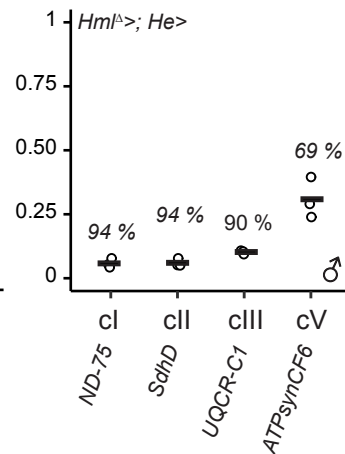

B

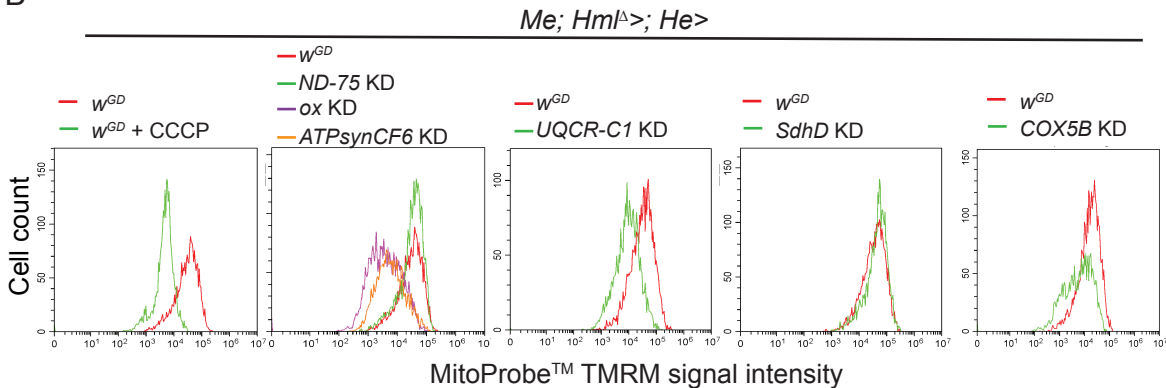

Supplement: Supplementary file 2 — Additional file 2: Fig. S1. Hemocyte-targeted OXPHOS RNAi constructs efficiently silence the OXPHOS gene expression and alter the mitochondrial membrane potential in hemocytes. (A-A’) Knockdown efficiencies of OXPHOS complex I (ND-75), cII (SdhD), cIII (UQCR-C1), cIV (COX5B) and cV (ATPsynCF6) RNAi constructs from the VDRC GD library (STable 1) in (A) female and (A’) male larval hemocytes. cIII gene ox and cIV gene COX5B knockdown efficiencies could not be tested due to the short gene length largely occupied by the RNAi hairpin structure. The percentages refer to the mean reduction in the mRNA levels in the OXPHOS knockdowns compared to those in the control hemocyte samples. (B) Histograms showing mitochondrial membrane potential in plasmatocytes measured by flow cytometry using the MitoProbe™ TMRM stain. [file 12915_2024_1858_MOESM2_ESM.pdf]

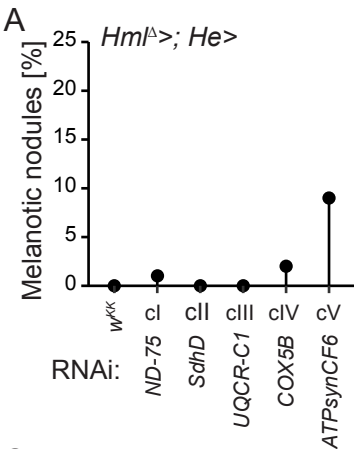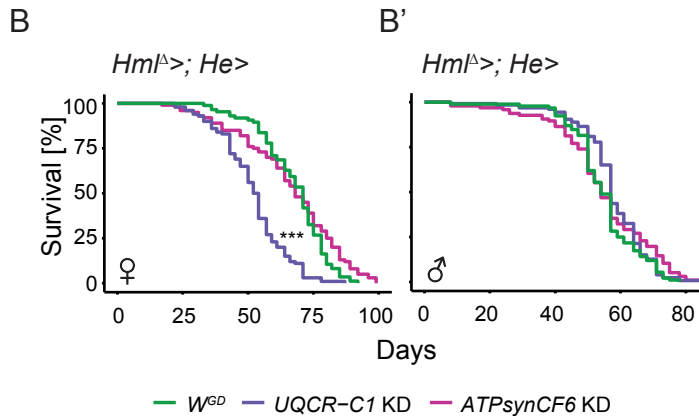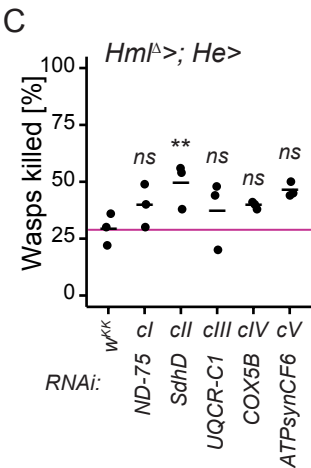

Supplement: Supplementary file 3 — Additional file 3: Fig. S2. Melanotic nodule prevalence and a detailed hemocyte analysis of OXPHOS complex I-V knockdown in RNAi strains from the KK library. (A) Quantification of melanotic nodules detected in the larvae (n = 100). (B-B’) Survival curves of female (B) and male (B’) UQCR-C1 and ATPsynCF6 knockdown flies and controls maintained at 25 °C. Log-rank test was used to compare differences in survival between groups. *** p < 0.001, n = 82–100. (C–C’’’’’) Quantification of total hemocytes and classification of hemocyte types based on eater-GFP and msn-mCherry expression when knocking down selected OXPHOS complex I-V genes in hemocytes (n = 30). Complex-specific target genes are: cI—ND-75, cII—SdhD, cIII—UQCR-C1, cIV—COX5B, cV—ATPsynCF6. (C–C’’) Total, plasmatocyte (pc) and activated plasmatocyte (act pc) counts. (C’’’-C’’’’’) Lamelloblast (lb), prelamellocyte (pre lc) and lamellocyte (lc) counts. The data were analyzed using a generalized linear model with a negative binomial distribution. Stars indicate the statistical difference of the OXPHOS gene knockdowns compared to the KK library background control (wKK). ns = not significant, * p < 0.05, ** p < 0.01, *** p < 0.001. [file 12915_2024_1858_MOESM3_ESM.pdf]

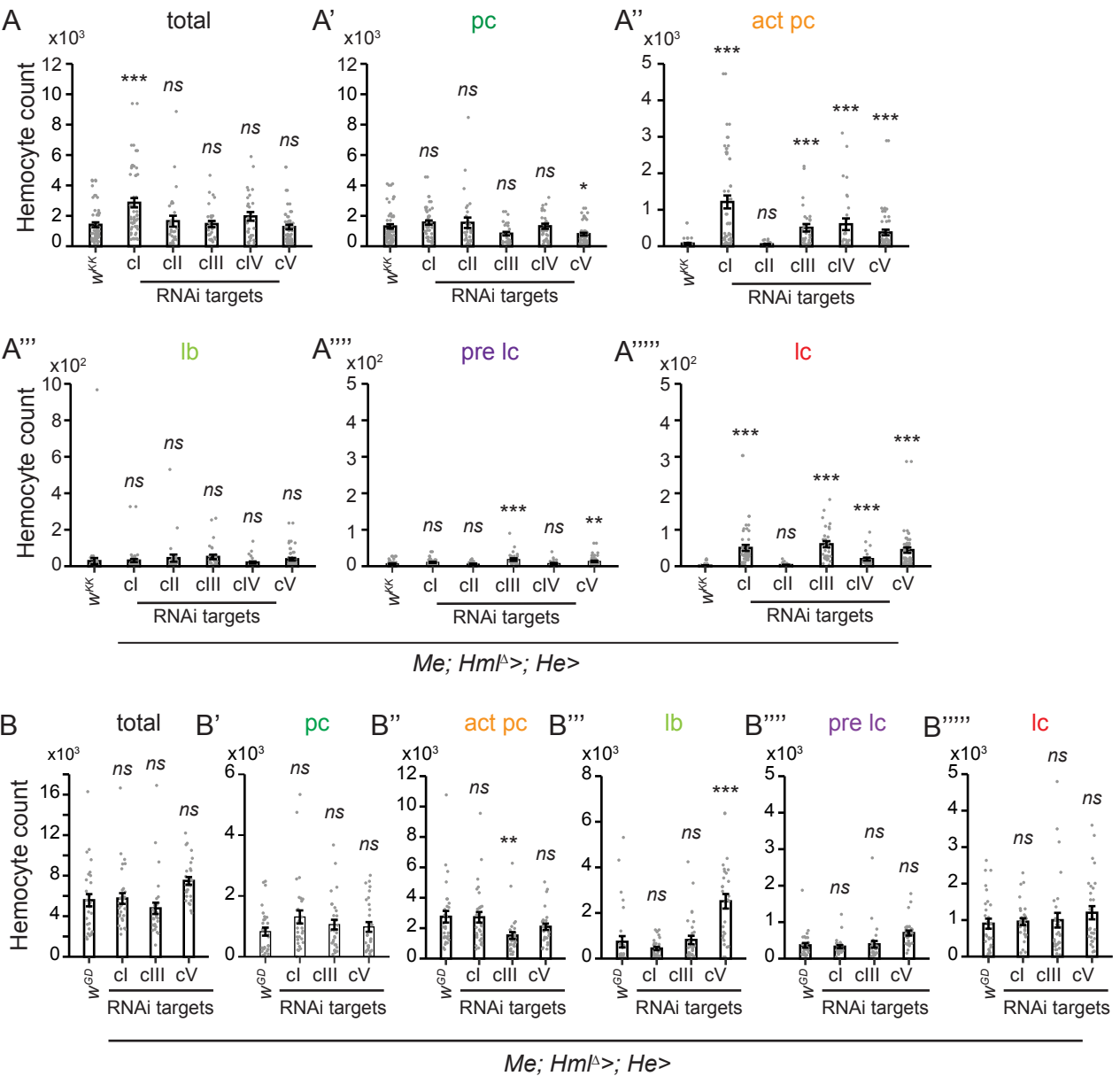

Supplement: Supplementary file 4 — Additional file 4: Fig. S3. Encapsulation response in RNAi strains from the KK library and hemocyte composition of selected GD library strains after infection. (A) Encapsulation efficiency assayed 48 h after L. boulardi infection in controls and after OXPHOS gene silencing in hemocytes using the RNAi strains from the VDRC KK library. (B-B’’’’’) Quantification of total hemocytes and classification of hemocyte types based on eater-GFP and msn-Cherry expression when knocking down selected OXPHOS genes in hemocytes (n = 30) and infecting the larvae with L. boulardi wasps. Complex-specific target genes from the GD library: cI—D-75, cIII—UQCR-C1 and cV—ATPsynCF6. (B-B’’) Total, plasmatocyte (pc) and activated plasmatocyte (act pc) counts. (B’’’-B’’’’’) Lamelloblast (lb), prelamellocyte (pre lc) and lamellocyte (lc) counts. The data were analyzed using a generalized linear model with a negative binomial distribution. Stars indicate the statistical difference of the OXPHOS gene knockdowns to the background control. ns = not significant, * p < 0.05, ** p < 0.01, *** p < 0.001. [file 12915_2024_1858_MOESM4_ESM.pdf]

A

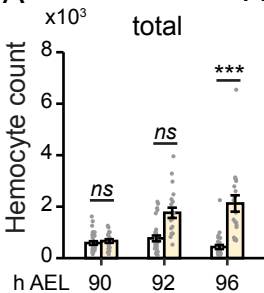

A'

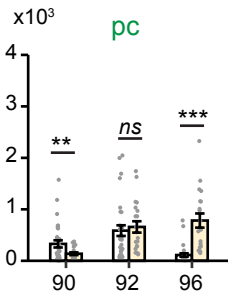

A''

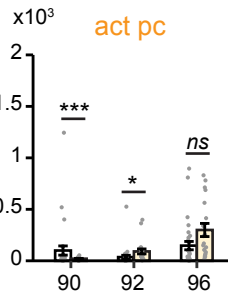

A'''

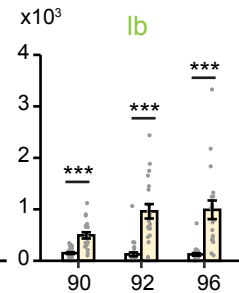

A''''

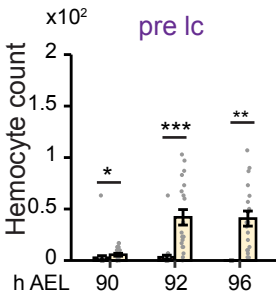

A'''''

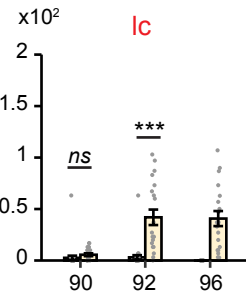

B

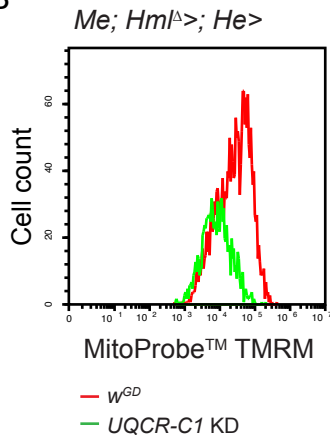

B'

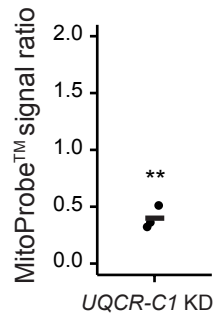

*Me; Hml<sup>Δ</sup>>; He>*

□ *w<sup>GD</sup>*    □ *UQCR-C1 KD*

Supplement: Supplementary file 5 — Additional file 5: Fig. S4. Hemocyte analysis of control and UQCR-C1 hemocyte knockdown larvae 90, 92 and 96 h after egg lay. (A-A’’) Total, plasmatocyte (pc) and activated plasmatocyte (act pc) counts. (A’’’-A’’’’’) Lamelloblast (lb), prelamellocyte (pre lc) and lamellocyte (lc) counts. The data were analyzed using a generalized linear model with a negative binomial distribution. (B-B’) Mitochondrial membrane potential in control and in UQCR-C1 knockdown hemocytes 90 h after egg lay was measured using the MitoProbe™ TMRM stain. The data were analyzed using one-sample t-test, comparing the ratio to a value of 1. ns = not significant, * p < 0.05, ** p < 0.01, *** p < 0.001. [file 12915_2024_1858_MOESM5_ESM.pdf]

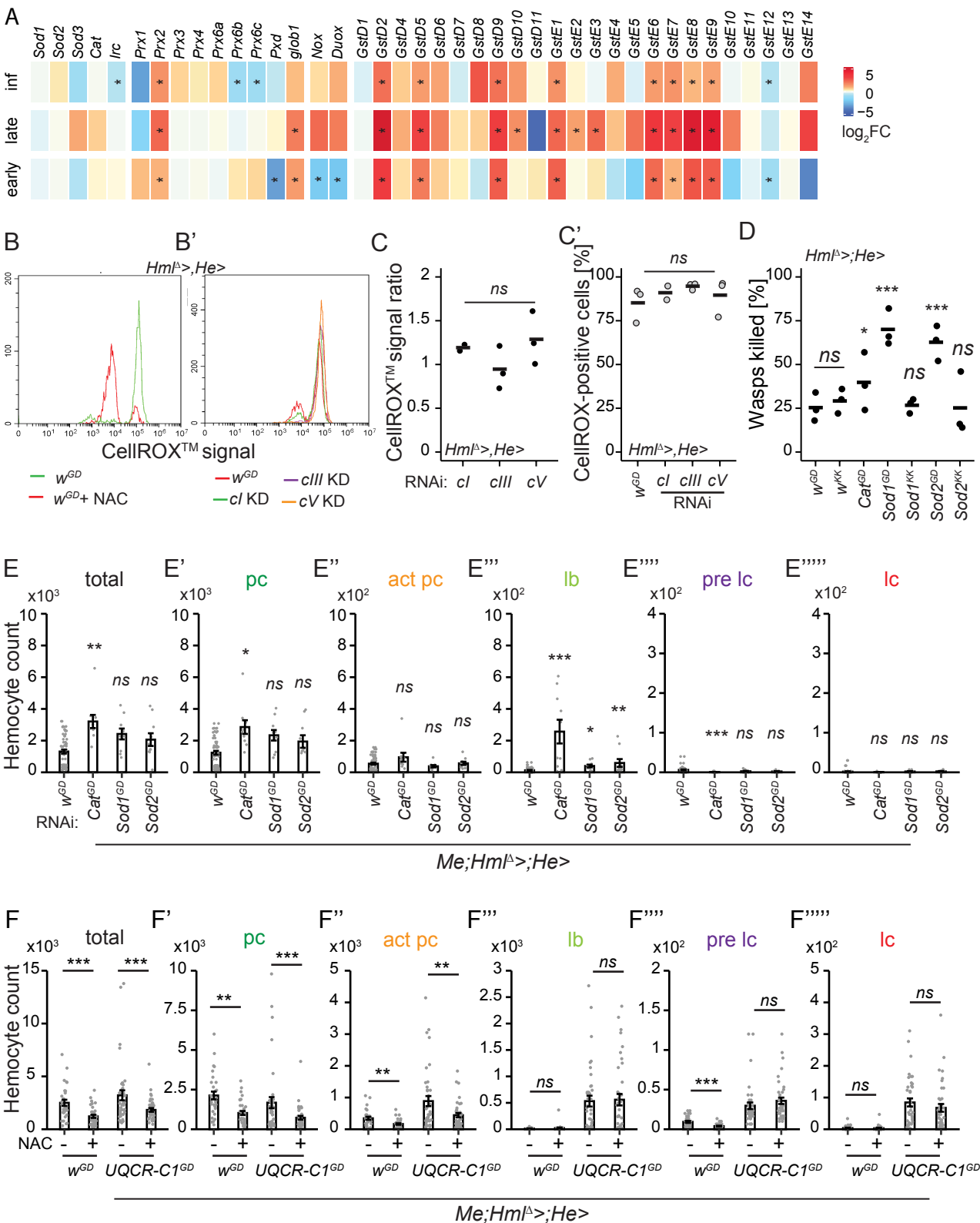

Supplement: Supplementary file 9 — Additional file 9: Fig. S5. ROS production is not crucial for the OXPHOS perturbation-related enhancement in immune response. (A) Heatmap showing the gene expression changes of genes related to reactive oxygen species (ROS) detoxification in early and late timepoints after UQCR-C1 knockdown and after wasp infection. (B-B’) Reactive oxygen species (ROS) were measured using the CellROX™ Green reagent. (B) CellROX™ Green signal in control hemocytes and in hemocytes obtained from N-acetyl cysteine (NAC)-fed larvae. (B’) CellROX™ Green signal in control hemocytes and in hemocytes with a knockdown of OXPHOS cI, cIII or cV genes. (C–C’) ROS levels were quantified as (C) a ratio of the CellROX™ Green signal in the OXPHOS complex knockdown hemocytes to that of the control hemocytes and as (C’) a proportion of hemocytes positive for the CellROX™ Green signal (n = 3, 5000–7000 hemocytes per replicate). cI = ND-75, cIII = ox, cV = ATPsynCF6. The ratios were analyzed using one-sample t-tests, comparing the ratios to a value of 1. The percentages were analyzed using a logistic regression with a binomial distribution. ns = not significant, * p < 0.05, ** p < 0.01, *** p < 0.001. (D) Control and antioxidant (AO) gene knockdown larvae were infected with L. boulardi wasps and the melanization response against the wasp eggs and larvae was assessed (n = 150). The data were analyzed using logistic regression with a binomial distribution. Replication was included as a random factor in the analyzes. (E-E’’’’’) Quantification of total hemocytes and hemocyte types classified based on eater-GFP and msn-Cherry expression when knocking down the AO genes in hemocytes of male larvae (n = 10). Of note, control samples are the same as in Fig. 2 C–C’’’’’ (n = 30). Asterisks indicate the statistical difference between the AO knockdowns and the control. Error bars indicate standard error of the mean. (F-F’’’’) Hemocyte quantification from control (wGD) and UQCR-C1 knockdown male larvae fed with norma [file 12915_2024_1858_MOESM9_ESM.pdf]

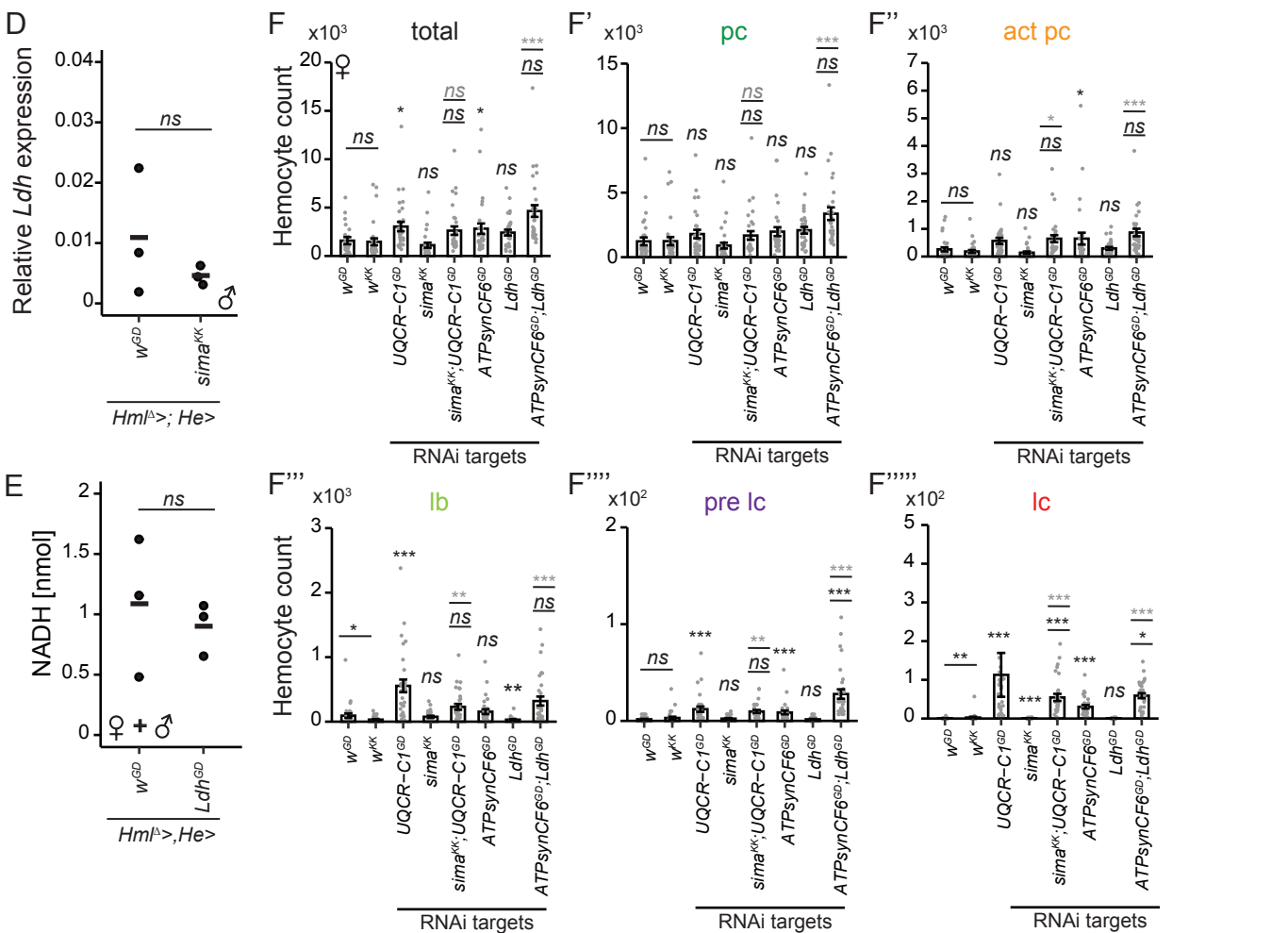 $Me; Hml^{\Delta}>; He>$

Supplement: Supplementary file 10 — Additional file 10: Fig. S6. Knocking down Ldh or sima in hemocytes does not affect hemocyte activation or differentiation. (A-B’) Expression of (A-A’) sima and (B-B’) Ldh was measured in hemocytes of 3rd instar OXPHOS knockdown (GD library) female and male larvae and is shown here normalized to the expression of the control gene His3.3B expression, and relative to the gene expression level in the hemocytes of control animals. cI—D-75, cII – SdhD, cIII—ox and cV—ATPsynCF6. Data on overall differences were analyzed using the Kruskal–Wallis test. When needed, pairwise differences were analyzed using the t-test, applying Bonferroni method to correct for multiple comparisons. ns = not significant, * p < 0.05, ** p < 0.01, *** p < 0.001. (C–C’) Knockdown efficiencies of sima and Ldh RNAi constructs in hemocytes of (C) female and (C’) male larvae normalized to His3.3B expression. Differences between the knockdowns and controls were analyzed using t-tests. (D) Ldh gene expression in control and sima knockdown hemocytes in males, normalized to His3.3B expression. (E) Production of NADH was measured as a readout of Ldh activity in control and Ldh knockdown hemocytes. Data shown is normalized to the amount of protein (µg/µl) to account for different amounts of starting material. Data in (D-E) were analyzed with t-tests. (F-F’’’’’) Quantification of total hemocytes and classification of hemocyte types based on eater-GFP and msn-mCherry expression in controls, in UQCR-C1 and sima single knockdown, as well as in sima; UQCR-C1 and Ldh; ATPsynCF6 double knockdowns (n = 30). Data from female larvae are presented. Two backgrounds strains (wGD and wKK) had very similar hemocyte profiles (similarly to males in Fig. 5C-C’’’’’), but with some statistically significant differences (underlined symbols above them). Not underlined significance symbols above the samples indicate their difference to the control. In the case of the double knockdowns, lower underlined symbol indicates the diffe [file 12915_2024_1858_MOESM10_ESM.pdf]

B:

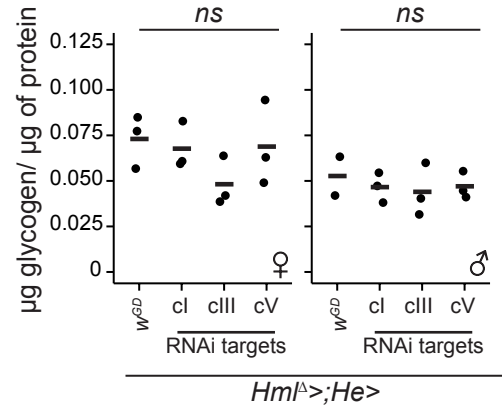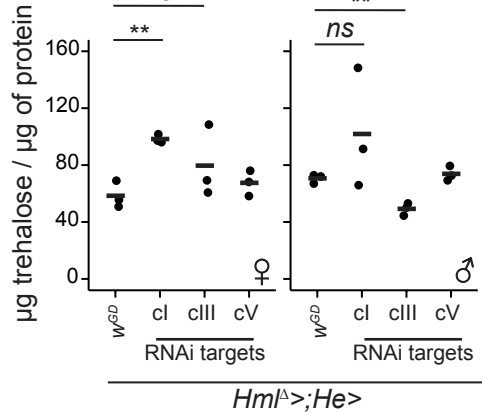

Supplement: Supplementary file 11 — Additional file 11: Fig. S7. Knockdown of the OXPHOS genes in hemocytes does not cause major changes in glycogen or trehalose levels. (A-A’) Storage sugar glycogen content normalized to protein content in (A) female and (A’) male larvae with OXPHOS knockdown in hemocytes. (B-B’) Content of circulating trehalose in hemolymph normalized to protein content in (B) female and (B’) male larval hemolymph with OXPHOS knockdown in hemocytes. cI—D-75, cIII—ox and cV—ATPsynCF6. The data were analyzed using pairwise t-tests, comparing each knockdown to the control (wGD). ns = not significant, * p < 0.05, ** p < 0.01, *** p < 0.001. [file 12915_2024_1858_MOESM11_ESM.pdf]
